# Laminar Analysis of Excitatory Local Circuits in Vibrissal Motor and Sensory Cortical Areas

**DOI:** 10.1371/journal.pbio.1000572

**Published:** 2011-01-04

**Authors:** B. M. Hooks, S. Andrew Hires, Ying-Xin Zhang, Daniel Huber, Leopoldo Petreanu, Karel Svoboda, Gordon M. G. Shepherd

**Affiliations:** 1 Janelia Farm Research Campus, Howard Hughes Medical Institute, Ashburn, Virginia, United States of America; 2 Department of Physiology, Feinberg School of Medicine, Northwestern University, Chicago, Illinois, United States of America; 3 The Solomon H. Snyder Department of Neuroscience, Johns Hopkins School of Medicine, Baltimore, Maryland, United States of America; Brain Mind Institute, Switzerland

## Abstract

Rodents move their whiskers to locate and identify objects. Cortical areas involved in vibrissal somatosensation and sensorimotor integration include the vibrissal area of the primary motor cortex (vM1), primary somatosensory cortex (vS1; barrel cortex), and secondary somatosensory cortex (S2). We mapped local excitatory pathways in each area across all cortical layers using glutamate uncaging and laser scanning photostimulation. We analyzed these maps to derive laminar connectivity matrices describing the average strengths of pathways between individual neurons in different layers and between entire cortical layers. In vM1, the strongest projection was L2/3→L5. In vS1, strong projections were L2/3→L5 and L4→L3. L6 input and output were weak in both areas. In S2, L2/3→L5 exceeded the strength of the ascending L4→L3 projection, and local input to L6 was prominent. The most conserved pathways were L2/3→L5, and the most variable were L4→L2/3 and pathways involving L6. Local excitatory circuits in different cortical areas are organized around a prominent descending pathway from L2/3→L5, suggesting that sensory cortices are elaborations on a basic motor cortex-like plan.

## Abstract 2

Optical and electrophysiological tools were used to map out the neural circuits within and between cortical layers in three different brain regions, and the results suggest regional specializations for sensory versus motor information processing.

## Author Summary

The neocortex of the mammalian brain is divided into different regions that serve specific functions. These include sensory areas for vision, hearing, and touch, and motor areas for directing aspects of movement. However, the similarities and differences in local circuit organization between these areas are not well understood. The cortex is a layered structure numbered in an outside-in fashion, such that layer 1 is closest to the cortical surface and layer 6 is deepest. Each layer harbors distinct cell types. The precise circuit wiring within and between these layers allows for specific functions performed by particular cortical regions. To directly compare circuits from distinct cortical areas, we combined optical and electrophysiological tools to map connections between layers in different brain regions. We examined three regions of mouse neocortex that are involved in active whisker sensation: vibrissal motor cortex (vM1), primary somatosensory cortex (vS1), and secondary somatosensory cortex (S2). Our results demonstrate that excitatory connections from layer 2/3 to layer 5 are prominent in all three regions. In contrast, strong ascending pathways from middle layers (layer 4) to superficial ones (layer 3) and local inputs to layer 6 were prominent only in the two sensory cortical areas. These results indicate that cortical circuits employ regional specializations when processing motor versus sensory information. Moreover, our data suggest that sensory cortices are elaborations on a basic motor cortical plan involving layer 2/3 to layer 5 pathways.

## Introduction

Sensation in the rodent vibrissal system relies on active whisking for interactions with the environment [1],[2]. Motor circuits control whisker movement, while sensory afferents collect information about contact with objects. Interactions between motor and sensory systems are necessary for object localization and identification [3]–[5].

Ascending sensory and descending motor pathways interact at multiple levels including the brainstem [6], thalamus [7], and cortex [8]. Three areas in the cerebral cortex are activated by whisker stimulation. Primary somatosensory cortex (vS1) responds with short latencies [9], whereas secondary somatosensory cortex (S2) and vibrissal motor cortex (vM1) respond 10–20 ms later [10]. These areas are also strongly interconnected in a bidirectional manner [8],[11].

In rodents, some of the cytoarchitectonic features of vM1, vS1, and S2 are area-specific, such as the presence of “barrels” in layer (L) 4 of vS1, and others are not, such as the presence of most cortical layers, including L1, L2/3, L5A, L5B, and L6 [12]. Here, to explore the synaptic organization of cortical circuits in these three areas, we used glutamate uncaging and laser scanning photostimulation (LSPS) to map the local sources of excitatory synaptic input to individual excitatory neurons in vM1, vS1, and S2. We recorded from postsynaptic neurons distributed across L2–6 (i.e., all the cortical layers that contain excitatory neurons) and, for each one, stimulated presynaptic neurons also distributed across L2–6. The collection of synaptic input maps for each area was analyzed to extract a laminar connectivity matrix representing the local pathways between excitatory neurons in each area [13],[14]. These connectivity matrices provide a quantitative survey of the interlaminar organization of local excitatory networks in each of these three cortical areas.

## Results

### Identification of Cortical Areas

**Figure 1:**
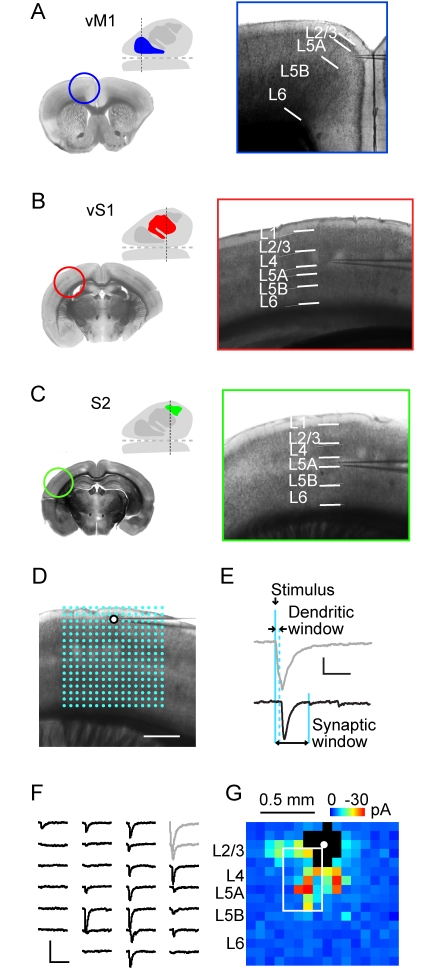
LSPS mapping in vM1, vS1, and S2. (A) Schematic of vM1 location (inset, blue) with approximate plane of coronal section indicated (dashed line). Anterior is to the left, and lateral is at top. Adjacent low power brightfield image (left) shows coronal section of vM1. Higher power brightfield image (right) shows vM1 slice used for recording. White lines indicate approximate cytoarchitectonic laminar boundaries. No division was evident between L2 and L3 or L5B and L6 in motor cortex. (B and C) Similar presentation for location and laminar boundaries in vS1 and S2. Sensory cortical boundaries were sharper than those in motor regions. Boundaries in S2 are similar to vS1. (D) Overlay of 16×16 LSPS stimulus grid (blue dots) on image of vS1 slice, for mapping inputs to a L2/3 pyramidal neuron. (E) Examples of dendritic (gray) and synaptic (black) responses. Vertical lines indicate photostimulus timing (at 0 ms) and windows for dendritic response detection (7 ms) and synaptic responses (50 ms). (F) Example of LSPS map traces (boxed region in G). Gray traces: responses with dendritic component. (G) Example map from a L2/3 neuron. Pixels represent mean amplitude over synaptic window. Black pixels: dendritic responses.

We identified vibrissal motor cortex (vM1), primary somatosensory (barrel) cortex (vS1), and secondary somatosensory cortex (S2) based on anatomical coordinates, cytoarchitectonic features, anatomical labeling experiments, and in the case of vM1, optical microstimulation mapping. vM1 (Figure 1A) was located in the posteromedial part of frontal agranular cortex, anteromedial to the barrel cortex [10],[15]–[17]. When anterograde tracers were injected into vM1, fluorescently labeled axons were observed in brainstem nuclei involved in whisker motor control (Figure S1) [18]. Furthermore, microstimulation mapping using channelrhodopsin-2 (ChR2) revealed that vM1 had the lowest thresholds for whisker movements (14) [19],[20]. vS1 (Figure 1B) was identified by the presence of cytoarchitectonic “barrels” in L4 [21]. S2 (Figure 1C) was located in dysgranular cortex, lateral to the barrel cortex [8],[10],[22]. Axons projected from vS1 to S2, and from S2 to vM1 and vS1 (Figure S1). These experiments enabled us to target our mapping experiments to specific cortical locations corresponding to vM1, vS1, and S2.

### Mapping Local Excitatory Pathways with Laser Scanning Photostimulation (LSPS)

We prepared coronal brain slices containing vM1, vS1, or S2 (Figure 1A–C) and used LSPS with glutamate uncaging [23]–[25] to map excitatory inputs to excitatory neurons (Figure 1D–G). We excited small clusters of neurons at each site in an array of locations while recording from individual excitatory neurons (Figure 1D,E), obtaining maps of local intracortical sources of excitatory input (Figure 1F,G).

**Figure 2:**
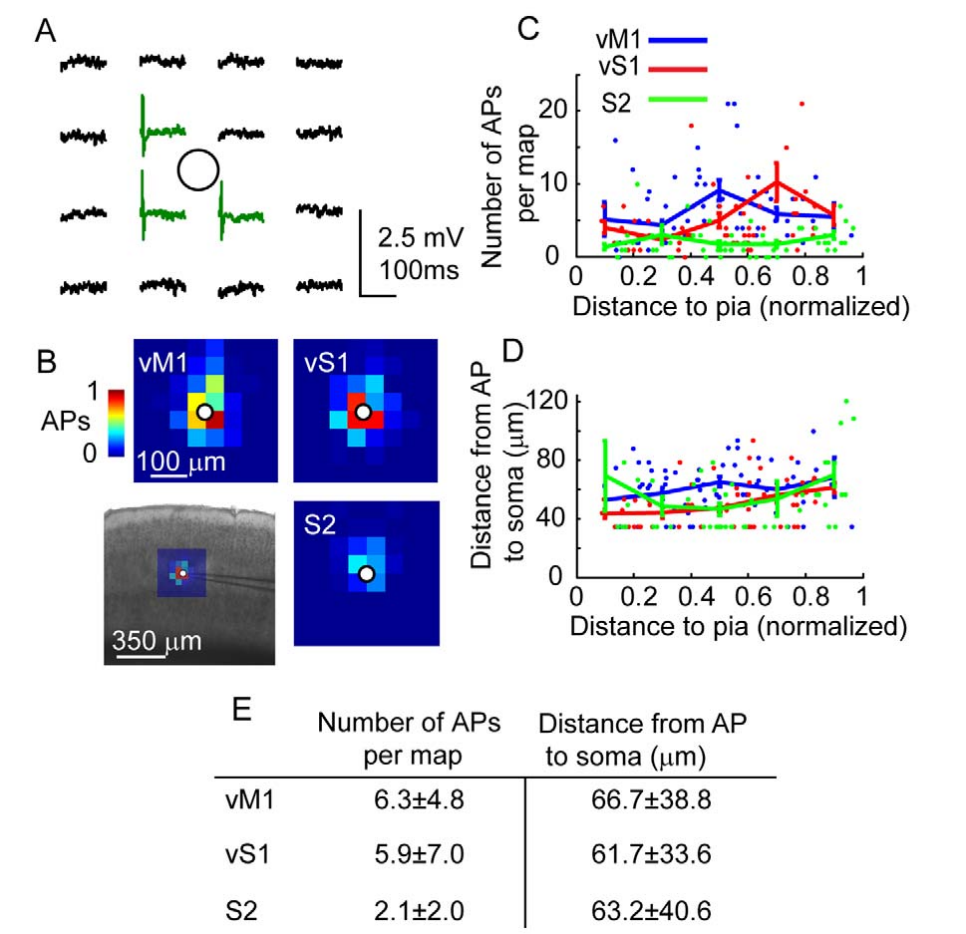
Photoexcitability of presynaptic neurons. (A) Excitation profile recorded from a L5B pyramidal neuron in vS1. Loose-seal recording was used to map the neuron's photoexcitable sites (grid: 8×8, 50 µm spacing, soma-centered). Central 4×4 region is shown. Circle: soma. Top of map is towards pia. Sites generating APs (green traces) were located peri-somatically. (B) Average excitation profiles for vM1 (left), vS1 (right), and S2 (bottom right) pyramidal neurons. Maps are soma centered. Bottom left, average vS1 excitation profile overlaid on slice image. (C) Number of APs per map per neuron, an estimator of the intensity of stimulation of neurons, plotted as a function of distance to pia. (D) Mean weighted distance from the soma of AP-evoking sites, an estimator of the resolution of stimulation of neurons, plotted as a function of distance to pia. For the grid used, the closest sites to the soma were at 35 µm. (E) Table summarizing excitability and spatial resolution for vM1, vS1, and S2.

To calibrate LSPS, we recorded in cell-attached mode from excitatory neurons, while uncaging glutamate on a grid around the cell (Figure 2A,B). The spatial distribution of action potentials (APs) evoked by uncaging (the “excitation profile”) provides a measure of the effective spatial resolution of photostimulation (Figure 2A,B). These data were used to estimate neuronal photoexcitability (Figure 2C) and the spatial resolution of LSPS (Figure 2D) for photostimulating neurons in different cortical layers and areas. Photostimulation-evoked APs always occurred in perisomatic regions (Figure 2B, Figure S2) with short latencies, and almost always as singlets. Stimulation of strong synaptic pathways, such as L4→L3 in vS1, did not cause APs in the target location (Figure S2), indicating that synaptic activity did not cause APs in neurons that were not directly photostimulated. Ultraviolet (UV) attenuation in scattering tissue causes photoexcitation to decline as a function of depth in the slice; consistent with this, excitation was not observed for neurons deeper than 100 µm (Figure S3) [26]. The total number of neurons excited per stimulus, estimated from the excitation profiles and measured densities of neurons (Figure 2 and Figure S4), was in the range 50–200, consistent with previous results [13],[26]. Only a small fraction of these neurons were synaptically connected to the recorded postsynaptic neuron [27].

An input map represents the aggregate functional synaptic connectivity between small clusters of presynaptic excitatory neurons at the stimulus locations and individual postsynaptic neurons. Pixels in input maps do not represent the strengths of unitary connections; rather, they measure average monosynaptic excitatory responses to a single uncaging event (see Text S1, Equations 1–4) [26]:
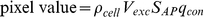
(1)where *ρ*_cell_ is the neuronal density at the point of uncaging (neurons/µm^3^), *V*_exc_ is the volume of excited neurons (µm^3^), and *S*_AP_ is number of APs fired per presynaptic neuron (AP/neuron). The average strength of a synaptic connection (*q*_con_) is calculated from equation (1). The collection of *q*_con_ for different neuronal populations defined by laminar location is the basis of connectivity matrices. We first present the mapping data for each area in the more familiar form of average input maps. In subsequent sections we summarize connectivity in laminar connectivity matrices, which take into account the parameters in equation (1).

### vM1 Maps

**Figure 3:**
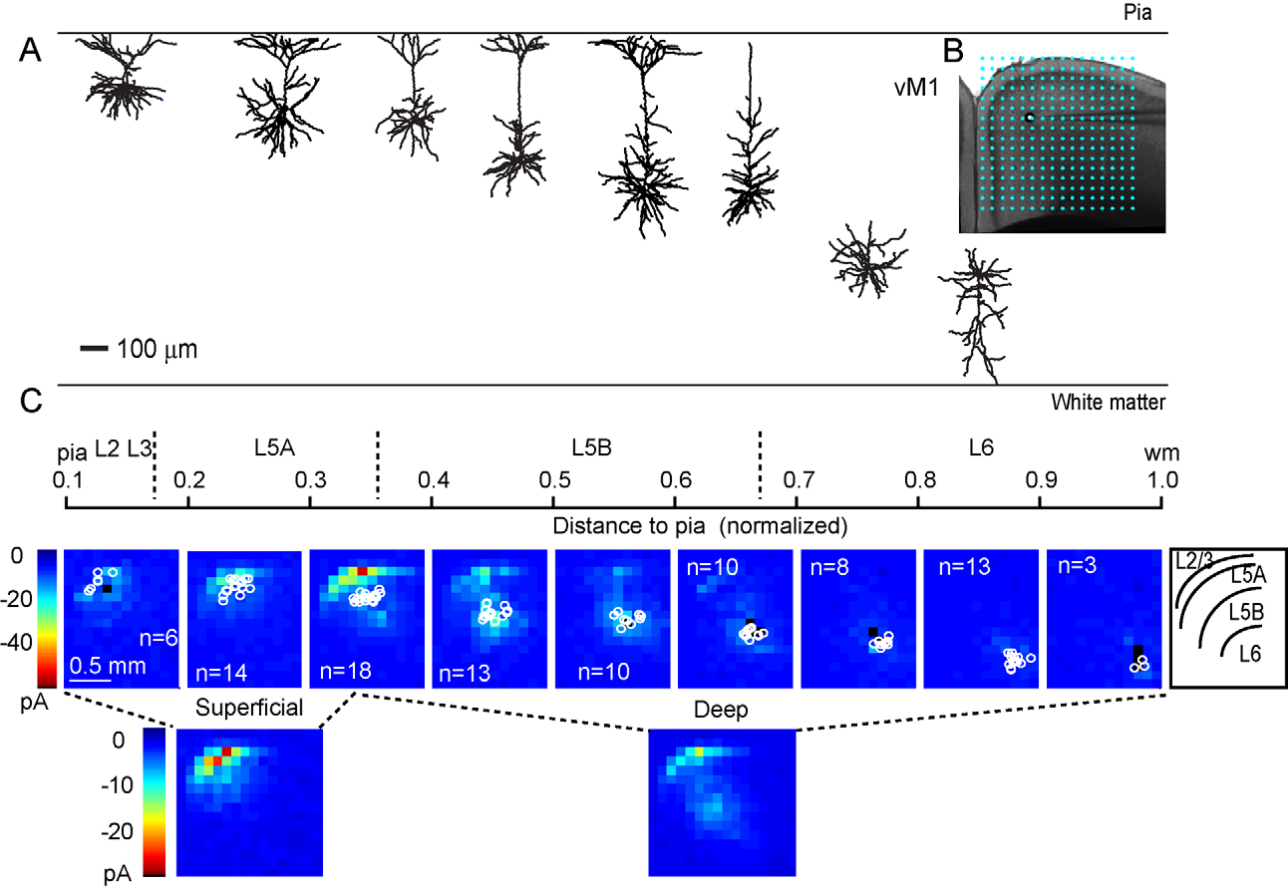
Average vM1 input maps. (A) Reconstructions of biocytin filled neurons in vM1. Neurons positioned according to radial distance (pia at top), and rotated to present the radial axis from pia to white matter as vertical. Axons not reconstructed. (B) Bright-field image of vM1, with overlaid LSPS grid (16×16 sites, 110 µm spacing), aligned to pia medially and superiorly. (C) Group-averaged input maps for neurons at different laminar depths. Normalized distances and approximate layers are indicated above the maps. Maps averaged by laminar depth in tenths (with no neurons in L1). Black pixels: dendritic response sites. Circles: somata. Points at map edges trimmed for display; color scale applies to all maps. Below, maps are averaged into groups of superficial (L2/3 and L5A) and deep (L5B and L6) neurons.

Unlike vS1, vM1 lacks a distinct granular L4. The superficial layers L2/3 and L5A are compressed, and deeper layers L5B and L6 are expanded, consistent with vM1's location at the crest of a cortical convexity [28]. In addition, L1 was thicker than in the other areas (Table 1). Both superficial and deep L5 neurons had dense basal dendrites and a single apical dendrite extending to L1, and L6 neurons had apical dendrites that did not extend to L1; in some cases, these were inverted pyramids (Figure 3A).

**Table 1:** Layer definitions based on morphometric measurements of layer boundaries in bright field images.

	Relative Depth			Actual Depth (in micrometers)		
	vM1	vS1	S2	vM1	vS1	S2
L1	0.10±0.01	0.09±0.01	0.11±0.01	160±16	128±16	137±10
L2		∼*0.13*	∼*0.20*			
L3	0.16±0.01	0.31±0.02	0.31±0.02	263±22	419±35	396±28
L4		0.46±0.02	0.45±0.02		626±42	572±29
L5A	0.34±0.02	0.54±0.02	0.55±0.01	570±38	733±45	688±27
L5B	∼*0.67*	0.74±0.03	0.76±0.02		1,006±55	952±38
L6	1	1	1	1,590±49	1,366±58	1,258±42
	*n* = 15	*n* = 62	*n* = 12	*n* = 15	*n* = 62	*n* = 12

Numbers correspond to lower borders of the corresponding layer. Cortical thickness was normalized (0, pia; 1, white matter) and presented ±SD. Italic numbers preceded by tildes indicate approximate borders where cytoarchitectonic distinctions were not strongly evident. We note that these measurements for vM1 were made at the mid-flexure (Figure 6B). The upper layers are relatively more expanded at more lateral locations.

We recorded from 95 excitatory neurons located in all layers (i.e., from upper L2 to lower L6) and mapped the local sources of excitatory synaptic input with LSPS using a stimulus grid that spanned vM1 (Figure 3B; Figure S5). We pooled neurons into groups by dividing the cortex into 10 equal distance bins; the top-most bin was empty, because L1 lacks excitatory neurons. We averaged the maps in each bin (Figure 3C). The strongest pathway was a descending projection, L2/3→ upper L5. Weaker ascending projections, within L5 and L5A→L2/3, were also found (Figure 3C). On average, neurons in the lower one-third (0.7–1.0) of vM1 showed weak inputs. However, individual neurons in this deeper range received strong inputs, but these tended to be spatially dispersed and sparse (Figure S5).

### vS1 Maps

**Figure 4:**
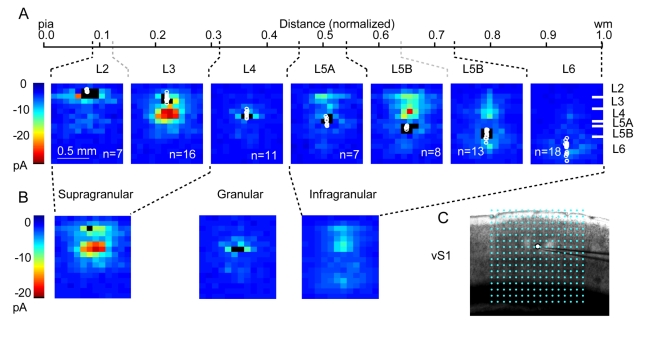
Average vS1 input maps. (A) Group-averaged input maps for neurons at different laminar depths. Maps are averaged by layer. Normalized distances and layer boundaries are indicated above the maps. On rightmost map, markers are given to indicate position of laminar boundaries between cortical layers. Black pixels: dendritic response sites. Circles: somata. Points at map edges trimmed for display; color scale applies to all maps. (B) Maps are averaged by supragranular and infragranular position and presented as above. (C) Bright-field image of vS1, with overlaid LSPS grid (16×16 sites, 90 µm spacing), aligned to pia superiorly and centered over the neuron horizontally.

We recorded from 80 excitatory neurons in vS1, using a different stimulus grid matched to the cortical thickness (Figure 4; Figure S6). In vS1, laminar boundaries were distinct, allowing pooling of cytoarchitectonically defined groups for binning (Table 1; Figure S8). The ascending L4→L3 pathway and the descending L2/3→L5 pathway were both prominent (Figure 4; Figure S6). Similar to vM1, L6 neurons had relatively weak inputs (mainly from L4). L4 neurons also showed little intracortical interlaminar input [29]. In addition, we further distinguished sub-layers within L2/3 and L5B based on patterns of connectivity observed in the input maps. For example, L2 constituted a narrow superficial layer of neurons lacking strong input from L4, but with input from L5A [30]. Binning with a simple three-layer scheme (‘supragranular-granular-infragranular’; Figure 4) conveyed the main feedforward local excitatory connections in vS1.

### S2 Maps

**Figure 5:**
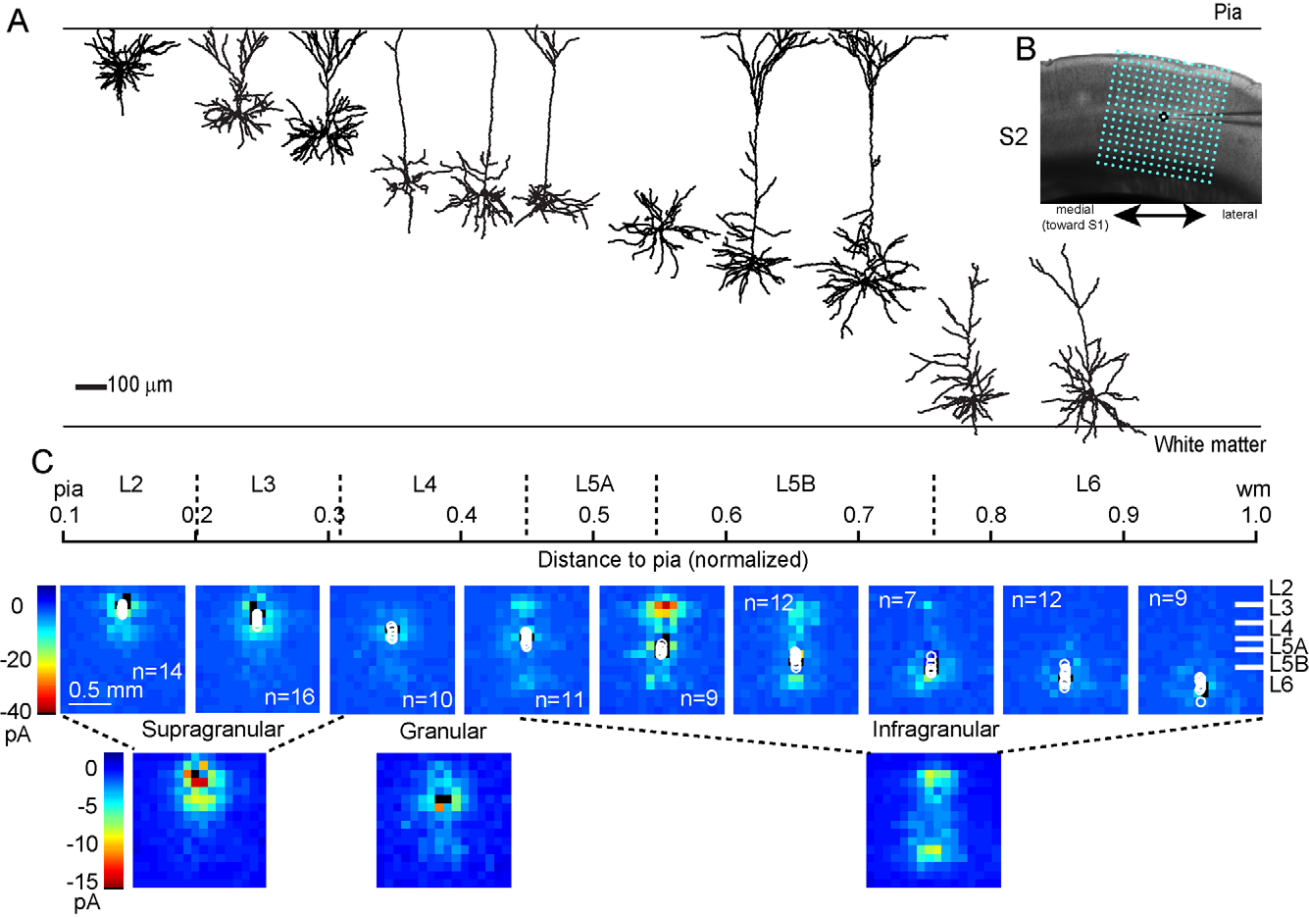
Average S2 input maps. (A) Reconstructions of biocytin filled neurons in S2. Neurons positioned according to radial distance (pia at top, with lines spaced every 0.2), and rotated to present the radial axis from pia to white matter as vertical. Axons not reconstructed. (B) Bright-field image of S2 at right (lateral) end of vS1, with overlaid LSPS grid (16×16 sites, 90 µm spacing), aligned to pia superiorly and centered over the neuron horizontally. S2 maps are aligned such that the medial side (S1) is to the left, as indicated below the image. (C) Group-averaged input maps for neurons at different laminar depths. Normalized distances and approximate layers are indicated above the maps. Maps averaged by laminar depth in tenths (with no neurons in L1). On rightmost map, markers are given to indicate position of laminar boundaries between cortical layers. Black pixels: dendritic response sites. Circles: somata. Color scale applies to all maps. Below, maps are averaged into groups of supragranular, granular, and infragranular neurons.

S2 abuts the lateral edge of vS1, where the barrel pattern terminates (Figure S1). The cytoarchitectonic layers appeared similar in S2 and vS1, except that the cortex was thinner and L5A thicker. L4 included neurons with a sparse apical dendrite, and neurons lacking an apical dendrite (Figure 5A). L5 neurons had many basal dendrites and an apical dendrite that ramified in L1; L6 neurons' apical dendrites did not extend above L4.

We recorded input maps for 100 excitatory neurons in S2 (Figure 5B,C; Figure S7). Similar to vS1, an ascending pathway to more superficial layers (L4→L3) was present but was not the strongest projection. Instead, the descending projection L2/3→L5 was predominant. L5 also received substantial ascending input from L6.

### Derivation of Connectivity Matrices

**Figure 6:**
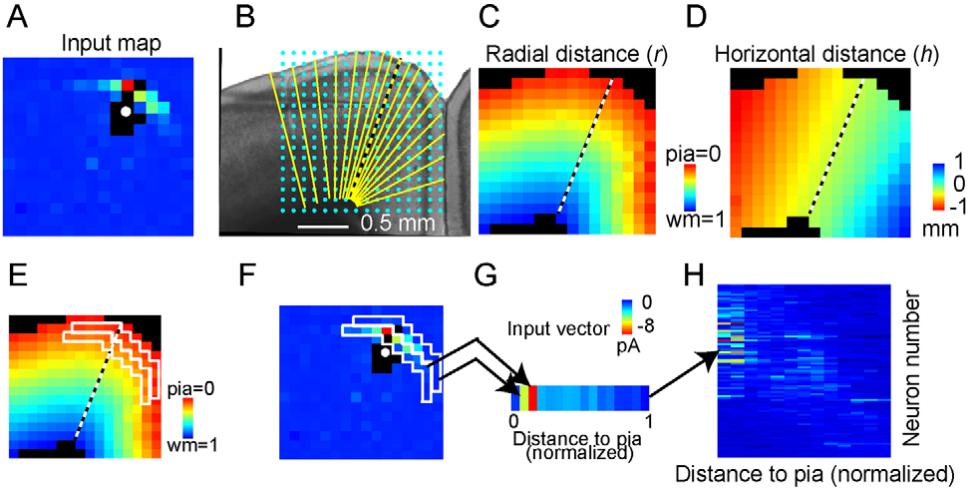
Converting input maps to input vectors for connectivity matrices. (A–D) Assigning coordinates of radial and horizontal distance to presynaptic locations in an input map: (A) Input map of a vM1 neuron. (B) Image of vM1 slice, overlaid with stimulus grid (blue dots), and radial spokes (yellow lines). Spokes were user-drawn along radial lines of the cortex. A central dashed spoke through recorded neuron's soma defines horizontal position *h*  =  0. These are used to measure stimulus grid locations (*x, y*) to transform them into (*h*, *r*) coordinates, where *h* is the horizontal arc distance (in µm) from the center spoke, and *r* is the normalized radial distance from the pia. Rainbow-like plots show interpolated maps of radial distance (C) and horizontal distance (D) for given points in an input map. (E–H) We selected points at a given presynaptic radial distance (two white boxes shown for adjacent superficial regions in E), within a limited horizontal range from the postsynaptic neuron. These regions were used to select points in the input map for binning purposes. By averaging the selected points in the input map at the given presynaptic depth (within the white boxes in F, for example), we converted input maps to input vectors (G). The postsynaptic radial distance for each recorded neuron was then used to place the input vectors in order, with vectors from superficial neurons in the top rows and deeper neurons in lower rows. By stacking the input vectors for every cell in a given cortical region, ordered by postsynaptic radial distance, a rough outline of the connectivity matrix can be presented (H).

Connectivity matrices represent local circuits in a compact manner [13],[14],[27],[31],[32]. Each element (*i, j*) in the matrix (**W** *_i,j_*) corresponds to the strength of a connection (*q*_con_; Equation 1) from the *j* ^th^ presynaptic location (along the rows) to the *i* ^th^ postsynaptic location (along the column). Distance is measured in normalized units along the radial directions (pia, 0; white matter, 1). Because of the curvature of vM1 at the cortical flexure (Figure 6A,B), we converted map data from the coordinates of the slice image (*x, y*) to coordinates corresponding to an unfolded cortex (*h, r*), where *h* is the horizontal distance along the laminar contour and *r* is the distance along the radial axis. Figure 6 provides a graphical illustration of the process of converting the pixels in an input map from *x-y* coordinates (Figure 6A), using a spatial transform defined on the basis of the radial structure of the cortex (Figure 6B), into *r-h* coordinates (Figure 6C,D).

**Figure 7:**
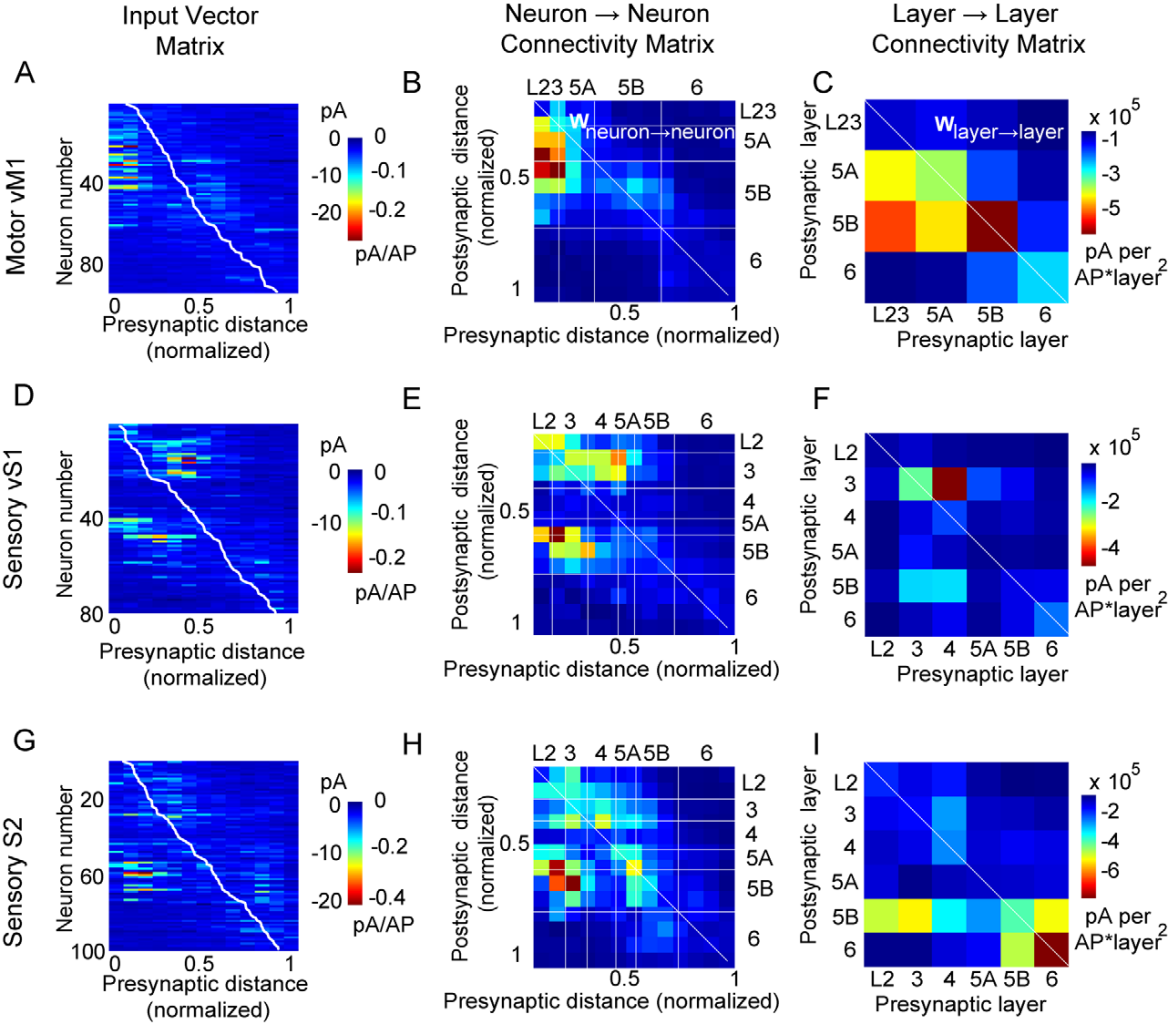
Connectivity matrices for vM1, vS1, and S2. (A, B, C) Connectivity matrices for vM1 (*n*  =  95 neurons). The input vector matrix (A) on left shows each neuron's input vector as a separate row, computed using the method in Figure 6. Rows are sorted by the normalized radial depth of the postsynaptic neuron's soma from L2/3 (top) to L6 (bottom). The white line indicates normalized radial depth for the given neuron in that row. Values given in pA per uncaging event, and binned by normalized distance from pia (1/14 of cortical depth, ∼90 µm). At middle, the neuron→neuron connectivity matrix (B) is given in pA per AP. Correction for presynaptic neuron density and number of APs (see Text S1) was applied to (A) to get the neuron→neuron connectivity matrix (B). White horizontal and vertical lines mark cortical layers; diagonal line marks within-layer connections. Top rows of the matrices (not shown) were empty, reflecting the absence of pyramidal neurons in L1. At right, the layer→layer connectivity matrix (C) is given in pA per AP per layer squared. Correction for presynaptic and postsynaptic neurons in each layer was applied to the neuron→neuron connectivity matrix (B) to derive the layer→layer connectivity matrix (C). This plot is binned by layer instead of by normalized distance from pia. (D, E, F) Connectivity matrices for vS1 (*n*  =  80 neurons). Left, individual neurons sorted as input vectors. Center, neuron→neuron connectivity matrix. Right, layer→layer connectivity matrix. (G, H, I) Connectivity matrices for S2 (*n*  =  100 neurons). Left, individual neurons sorted as input vectors. Center, neuron→neuron connectivity matrix. Right, layer→layer connectivity matrix.

This approach allowed us furthermore to convert input maps into vectors, by averaging input across the horizontal dimension (*h*) at a given presynaptic radial distance (*r*) into bins (Figure 6E–G; a similar analysis in the horizontal dimension is given in Figure S9). This is identical to averaging along the rows of input maps, except that it takes into account the curvature of the cortex. One neuron's input vector (Figure 6G) thus represents the inputs to one neuron from different laminar locations; i.e., the horizontal dimension has been collapsed. Each neuron was also assigned a postsynaptic radial distance. This allowed us to group all the input vectors and then sort them by the postsynaptic neuron's depth in the cortex (Figure 6H). Stacking the vectors on top of each other, sorted by depth, provided a raw connectivity matrix, **W** *^raw^*(*r*_post_, *r*_pre_), describing connectivity between neurons at different locations along the radial axis (Figure 6H, Figure 7A). The rows in such a connectivity matrix represent synaptic input to a particular laminar location, and the columns represent synaptic output from that laminar location. Intralaminar connections lie along the main diagonal. We note that intralaminar connectivity was undersampled because of direct excitation of the postsynaptic neurons' dendrites.

In addition to deriving matrices based on the collections of input vectors (Figure 7A,D,G), we further analyzed the data in terms of the excitation parameters given in equation (1). To compute the average connectivity matrix at the level of individual neurons (**W** *^neuron^*), we binned the data and applied correction factors to derive the strength of input per presynaptic neuron per AP. We divided the connection strength in the raw connectivity matrix by the mean number of APs per uncaging event at the presynaptic region (Figure 7B,E,H; Figure S10; Text S1) and the number of presynaptic neurons stimulated. The number of stimulated neurons was obtained from measurements of *ρ*_cell_ (Figure S4) and *V*_exc_. To compute the connectivity matrix at the level of cortical layers (**W** *^layer^*) we multiplied the neuron→neuron connections by the number of presynaptic and postsynaptic neurons per layer (Figure 7C,F,I; a detailed calculation is illustrated in Figures S11 and S12). Values for all connectivity matrices are provided in Table S1 and Dataset S1.

## Discussion

We used glutamate uncaging and LSPS to map local synaptic connections among excitatory neurons in mouse vM1, vS1, and S2, three cortical areas centrally involved in vibrissa-based somatosensation. From single cell input maps recorded at different cortical depths, we derived connectivity matrices that compactly describe the local network. Our main findings were that vM1 contains a strong pathway from L2/3 to upper L5; that vS1 and S2 contain two strong pathways, corresponding to L4→L3 and L2/3→L5; and that S2 contains these plus pathways between L6 and L5B.

### Connectivity Matrix Descriptions of Cortical Circuits: Neuron→Neuron and Layer→Layer

The connectivity matrix description allows us to directly contrast local circuits in different cortical regions. The elements (pixels) in the neuron→neuron connectivity matrices, **W** *^neuron^* (Figure 7B,E,H), represent the mean strength of postsynaptic response in a single neuron extrapolated to a single presynaptic AP in a single cell of the indicated layer (*q*_con_). Pixel values were 10–100 times lower than typical unitary EPSCs, reflecting both the generally low probability of connections between excitatory neurons in cortical circuits (typically 0.1–0.2) [27],[33]–[35], and the fact that the current amplitude in the maps represents a mean over 50 ms rather than the peak of the EPSC.

In contrast, the elements in the layer→layer connectivity matrices, **W** *^layer^* (Figure 7C,F,I), represent the average strength of connections extrapolated to the entire projection from one layer to another. The **W** *^layer^* matrices differ from the **W** *^neuron^* matrices in that they enhance thicker and more neuron-dense layers and diminish thinner and less neuron-dense layers. For example, because in vS1 the L5A is thin (Table 1) and both L5A and L5B are low in neuronal density (Figure S4), the projections to and from L5, such as L5A→L2/3 and L2/3→L5B, are relatively strong at the level of neuron→neuron connectivity (Figure 7E) but relatively weak at the level of layer→layer connectivity (Figure 7F). Interestingly, in rat vS1 the L4→L2/3 projection is functionally weak compared to the structural density of L4 axons and L2/3 dendrites, while the converse holds for the L5A→L2/3 projection [36]. Our results here show how weak neuron→neuron connections may be strong in aggregate at the layer→layer level. Further structure-function analyses will be required to determine whether it is generally the case that larger and more neuron-dense layers have weaker neuron→neuron but stronger layer→layer projections.

### Major Features of Connectivity Matrices in the Three Areas

The connectivity matrix representations of vM1 show strong descending projections from L2/3→upper L5 (Figure 7A–C), similar to the forelimb area of mouse M1 [13],[14],[37]. This input straddled the L5A/B border. L5B received an additional hotspot from itself, which appeared strong when considered as an entire layer (Figure 7C). The deepest one-third of vM1 (consisting mostly of L6) had weak inputs and outputs.

The vS1 excitatory circuits were more complex (Figure 7D–F). The major ascending pathway from L4→L3 was paralleled by an ascending component from L5A. The high cell density in L4 made the L4→L3 connection prominent in the laminar analysis (Figure 7F). Another prominent projection was from L2 and L3 to L5A and L5B; inputs originating in more superficial regions of L2/3 targeted relatively more superficial regions of L5A/L5B (note the diagonal shape of the L2/3→L5 hotspot in Figure 7E). On a neuron→neuron basis, the L3→L5B connection was stronger than L4→L3, although the layer→layer analysis showed a reduction in cell density relative to L4. L2 received input from L3, and weaker input from L5A. However, L2 was thin and thus contributed little to **W** *^layer^*. As in vM1, deep layers had weak inputs and outputs.

**Figure 8:**
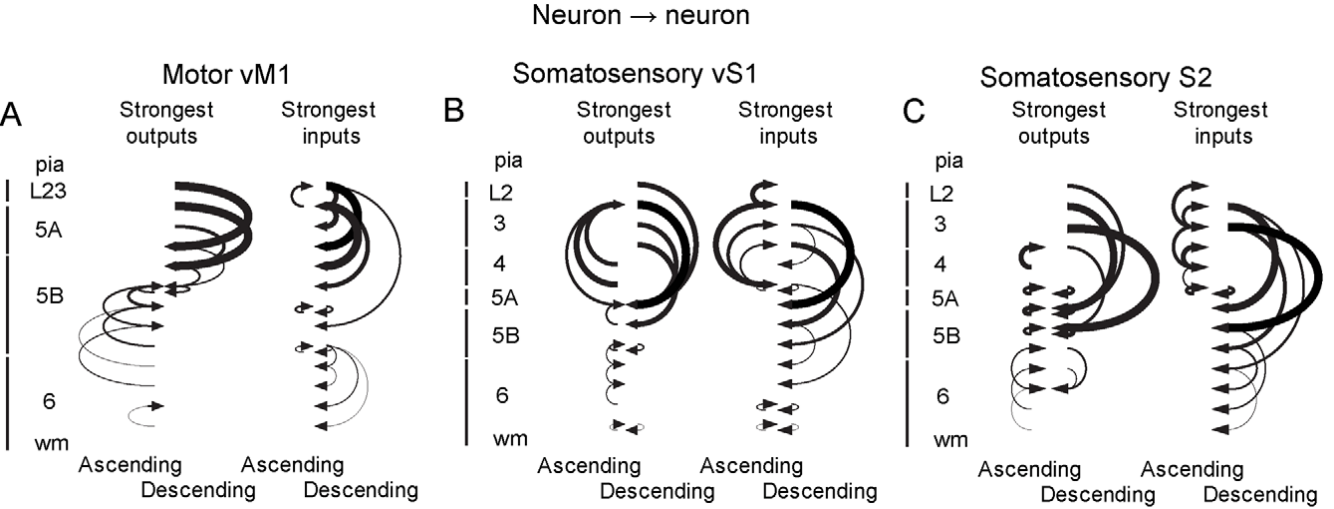
Laminar wiring diagrams of three vibrissal-related areas. (A) vM1. Using the neuron→neuron connectivity matrix, input-output wiring diagrams of cortical circuits are shown. Layers are indicated, with pia and superficial layers at the top, and neurons are divided into evenly spaced bins. Left, the strongest outputs at each bin. Only the single strongest projection from each bin is shown. Ascending pathways are shown on the left; descending connections on the right. Right, the strongest inputs at each radial depth. Only the single strongest connection to each bin is shown. Ascending inputs are shown on the left; descending inputs on the right. The arrow thicknesses indicate the relative strengths of connections. (B and C) Wiring diagram of vS1 and S2 displayed in similar fashion.

In S2 (Figure 7G–I), an ascending L4→L2/3 pathway and descending L3→L5 pathway were present. Neurons on the L5A/L5B border also showed strong intralaminar connections. The L6 output evident in the input maps (Figure 5; Figure S7) also supplied potent input to L5B. Although not as strong at the single cell level, the entire L6 excited L5B as much as L3 (Figure 7I). L6 was enhanced in S2 relative to other regions as both a source of synaptic output and a recipient of synaptic input, due to the relatively high density of neurons (Figure S4) and their relatively low photoexcitability (Figure 2C–E). The functional connectivity in the local excitatory circuits of all three regions is simplified into quantitative laminar wiring diagrams (Figure 8).

### Limitations in the Derivation of Connectivity Matrices

LSPS with glutamate uncaging simultaneously excites a group of presynaptic neurons, while the postsynaptic response is measured. To derive average connection strength per neuron (*q*_con_), the number of excited neurons needs to be estimated, based on the excitability (*S*_AP_), neuron density (*ρ*_cell_), and excitation volume (*V*_exc_) at the uncaging location (Equation 1). The accuracy of the estimate of *q*_con_ is limited by our measurement of *ρ*_cell_ and neuronal excitation (*S*_AP_, *V*_exc_; Text S1 Equations 3–4): Measurements of neuronal density vary by a factor of two [27],[38],[39]. Although excitation profiles give a direct measure of evoked APs in brain slices under the relevant recording conditions (Figure 2), excitation varies across neurons and somewhat across cortical areas, and decreases with depth in the slice; these effects together introduce uncertainty roughly on the order of a factor of two (Figure S3). Despite these uncertainties, our estimates of *q*_con_ are broadly consistent with those derived from pair recordings (Figure S13).

Because LSPS excites many neurons, this strong stimulus allows weak pathways to be detected. However, the average connection strength, *q*_con_, reflects both the connection probability and unitary connection strength:
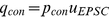
(2)It is therefore not possible to separate connection probability and unitary connection strength directly. Furthermore, *p*_con_ is inversely related to the horizontal separation between cell pairs [34]. LSPS averages inputs from a range of presynaptic locations with varied horizontal offset. For each cell class, a broad distribution of *p*_con_ values contributes to LSPS maps.

In addition, by computing the average connection strength, we average out the underlying distribution of unitary connection strength, which is a skewed distribution of numerous weak and a few strong connections [27],[33]. This inherent averaging also makes LSPS insensitive to certain non-laminar aspects of cell-type specificity in cortical connectivity [14],[33],[35],[40]–[43].

### Comparisons with Previous Studies of vS1 Connectivity

Comparison of our neuron→neuron connectivity matrix with a pair-recording study [27] reveals qualitative similarities (Figure S13). After both methods are corrected to similar units (peak amplitude in pA/AP), the general shape of the connectivity matrix and values for neuron→neuron connectivity are similar. The major interlaminar pathways are L2/3→L5 and L4/5A→L2/3. However, local intralaminar connections are underestimated in our data set due to direct responses to uncaging. Furthermore, descending projections from L4→L5A and from L5A→L5B may be underestimated in LSPS relative to pair recording due to exclusion of direct responses along the apical dendrite of the postsynaptic neuron (see L5A and L5B maps in Figure S6). Under-sampling of connected pairs in low-*p*_con_ pathways, such as L4→L6, may account for differences from LSPS, where many L4 neurons are excited during each L6 recording. Lastly, L2 connectivity differs in part because of differences in the definition of this layer.

### Inter-Areal Comparisons: Ascending Pathways to Supragranular Layers

We compared the matrices for the four areas so far studied, vM1 (present study), the forelimb region of somatic M1 [13], vS1 (also the present study) [27], and S2 (present study). Overall, the main differences are attributable to the presence of a distinct granular layer in somatosensory cortex. Specifically in vS1, L4 outflow contributed strongly to the connectivity matrix. L4→L2/3 is also a major pathway in rodent V1 [44]. In S2, the local excitatory circuit differs from vS1 most prominently in that the L4→L3 pathway is reduced. LSPS analyses of auditory cortex circuits have found L4→L2/3 inputs [45],[46], which is adjacent to S2. However, ascending pathways were not unique to vS1, as a similar but weaker L3/5A→L2/3 pathway was prominent in forelimb M1, and present but weaker still in vM1 (Figure 3C and Figure S5C, leftmost panels). The upward compression of layers in vM1, typical of cortical convexities [28], may be why L3/5A→L2 was less distinct in vM1 than in forelimb M1 (e.g., it was more prone to masking by dendritic responses of L2 neurons). However, inspection of individual maps and traces (Figure S5C) showed that these ascending pathways were present for some L2 neurons.

### Inter-Areal Comparisons: Descending Pathways to Deep Layers

A second main interlaminar hotspot in vS1 was the descending pathway(s) L2/3→L5, which was the predominant hotspot in the two motor areas. We noted that this pathway was present in all three cortical regions studied here and was similarly prominent in somatic M1 [13]. Indeed, it was the predominant pathway in S2. Thus, a strong supragranular to infragranular descending connection emerged as a common element of local cortical circuits examined here. Superficial L5B neurons and deep L5A neurons at the laminar border were most strongly activated, suggesting that the cytoarchitectonic boundaries identified do not correspond well with functional gradient within L5. Perhaps an alternative molecular marker, such as Etv1 (Figure S8), better denotes this functional division.

### Inter-Areal Comparisons: Involvement of Deep Neurons in Local Circuit Function

In three of the four areas, L6 neither received nor sent strong projections (but vS1 neurons in L6 received a weak projection from L4). L6 output is provided by an ascending connection to L4 in cat visual cortex [31],[32],[47] but was absent or reduced in all vibrissal areas we studied. L6→L4 projections studied in mouse somatosensory and auditory cortical areas have “modulator” rather than “driver” properties, including paired pulse facilitation [48]. Although deeper neurons tend to have relatively small dendritic arbors [49], which may account for a reduction (but not absence) of inputs, this difference in arbor size is not of sufficient magnitude to account for the paucity of inputs. Similarly, the paucity of outputs was not due to lack of photoexcitability of these neurons. Channelrhodopsin-assisted circuit mapping experiments [50] have shown that the supragranular layers indeed connect preferentially to upper rather than lower infragranular neurons. Thus, the lack of inputs was not due simply to slice-related artifacts such as severing of pathways. Consistent with weak local inputs, in vivo recordings in cat motor cortex suggest that a large number of L6 neurons are virtually silent, even during motor activity [51]. Thus, the sources and modes of excitation for L6 neurons remain to be determined [49],[52]. However, L6 was more engaged in local circuits in S2, supplying a measurable output to L5A and L5B and to other L6 neurons. In addition to input from L5B, L6 neurons in S2 collected inputs from a wide horizontal distance, sometimes >300 µm (Figure S7B,C at right). Thus, S2 may be better suited for studying L6 function.

### Quantitative Comparison of Cortical Microcircuits

**Figure 9:**
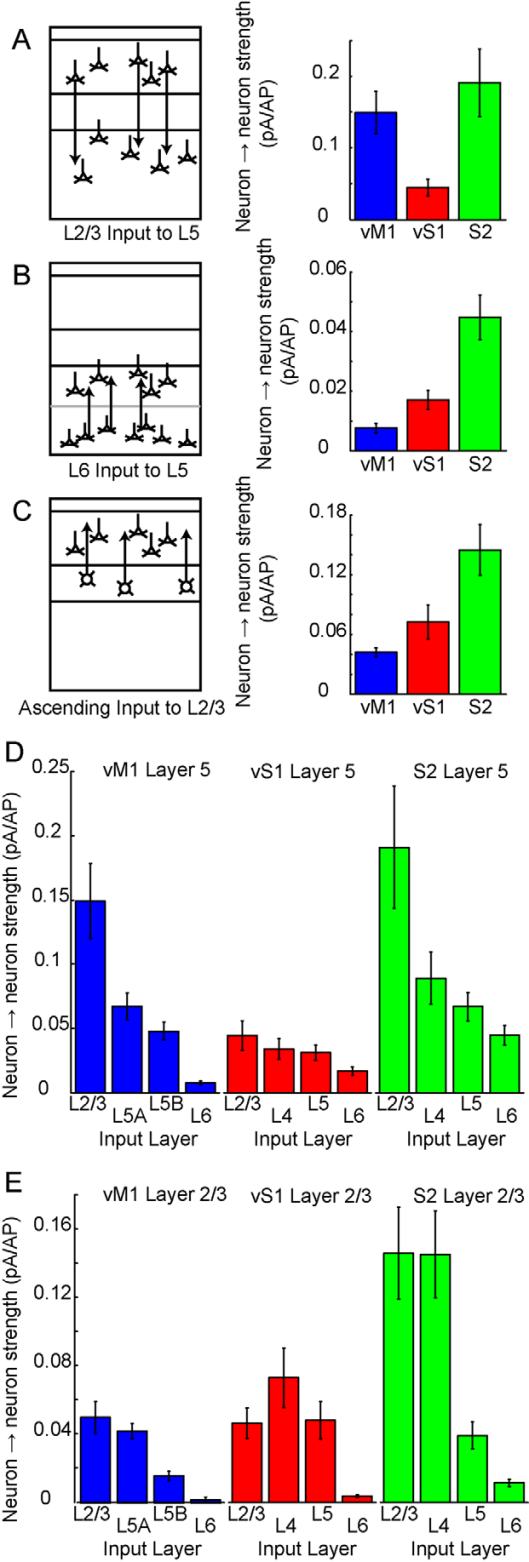
Quantitative comparison of neuron→neuron connectivity. (A) Neuron→neuron pathway strength presented for vM1, vS1, and S2. Left, schematic of descending pathway from L2/3→L5. Cartoon is based on vS1 layers. Right, quantification (mean ± SD) of neuron→neuron pathway strength. Measurements of connection strength from input maps, of excitability in each cortical area, and of neuron density were resampled 10,000 times in a bootstrap analysis to determine variation (standard deviation). (B and C) Similar presentation for the ascending pathway from L6→L5 and L4→L2/3. For vM1, ascending input from L5A→L2/3 is presented in (C). (D) Quantification of all inputs to L5, presented on the same axis for comparison. (E) Quantification of all inputs to L2/3, presented on the same axis for comparison.

One major difficulty in making a comparison of connectivity between two cortical areas is selecting the laminar position of pre- and postsynaptic neurons for the comparison. Is it better to compare identical relative laminar depths between cortical areas, not accounting for the decreased thickness of superficial layers, and increased thickness of deep layers, in motor areas? How shall we treat the presence or apparent absence of a distinct layer 4? We present a direct quantitative comparison of three major areas identified in our study, based on cytoarchitectonic laminar divisions (Table 1, Figure S8) (Figure 9). In vS1 [53] and vM1 (Tianyi Mao, BMH, GMGS, KS, unpublished observations) these layers correspond to distinct cell types with different projection patterns. The descending projection from L2/3→L5A/B was prominent in all areas, but the strength of the pathways at a neuron→neuron level varied by a factor of four between the areas. Ascending projections from middle layers to superficial ones (L4→L2/3 in vS1 and S2; L5A→L2/3 in vM1 for comparison) were also present in all regions but were the least prominent in agranular vM1. Lastly, the L6→L5 projection identified in S2 was more than twice as strong at the neuron→neuron level than in vS1 (and the difference was greater with vM1). Our approach provides a defined framework for measuring similarities and differences between cortical microcircuits in a quantitative manner.

## Materials and Methods

### Terminology for Cortical Axes

We use the term *radial* to refer to the axis defined by the apical dendrites of pyramidal neurons; this axis is approximately normal to the cortical surface. *Normalized radial distance* is along the radial axis, bounded by the pia and the white matter, where pia  =  0 and the L6/white matter border  =  1. *Vertical* is synonymous with *radial*. *Horizontal*, or *lateral*, refers to planes normal to the radial axis, approximately parallel to *layers*, or *laminae* (Figure 6). *Oblique* refers to off-axis interlaminar connections.

### Slice Preparation

Mice were decapitated at postnatal day 20–25 under isofluorane anesthesia, and the brain rapidly placed in ice cold choline solution (in mM: 110 choline chloride, 25 NaHCO_3_, 25 D-glucose, 11.6 sodium ascorbate, 7 MgCl_2_, 3.1 sodium pyruvate, 2.5 KCl, 1.25 NaH_2_PO_4_, 0.5 CaCl_2_). Coronal brain slices (300 µm) were cut (Microm HM 650V), incubated 30 min at 37°C in oxygenated ACSF (in mM: 127 NaCl, 25 NaHCO_3_, 25 D-Glucose, 2.5 KCl, 2 CaCl_2_, 1.25 NaH_2_PO_4_, 1 MgCl_2_), and maintained in a holding chamber at 22°C for up to 5 h during recording. For vM1 slices, the brain was pitched upward ∼10° to optimize alignment with the radial axis of vM1, and slices ∼0.7–1.3 mm anterior to bregma were used; for vS1 and S2 slices, the brain was cut coronally, and slices ∼1–2 mm posterior to bregma were used (Figure 1A,B). To determine the optimal slice angle for each area, we used the appearance of the intact apical trunk at high magnification to select slices for recording and avoided those sections where the apical dendritic trunk was at an angle with respect to the slice plane. Thus, only one or two sections per animal could be used for recording. Separate experiments in our laboratory using the photostimulation methods in vS1 [42] and vM1 (unpublished data) measure input to L1 dendrites of L5 pyramidal neurons, confirming the apical trunk is intact using this slice angle. We added biocytin to visualize a subset of dendritic arbors, some of which are reconstructed in Figure 3 and Figure 5. These neurons appeared radially symmetric, with arbors ranging from 300–500 µm in diameter. Since the neurons were 50–100 µm deep in the slice, a portion of the apical and basal dendrites are truncated by slicing and the deep half of the arbor is intact.

### Electrophysiology

Recordings were performed at room temperature (22°C) in ACSF. Neuronal excitability was reduced by increased divalent ions (4 mM CaCl_2_ and 4 mM MgCl_2_), and NMDA receptor blockade with 5 µM 3-((R)-2-carboxypiperazin-4-yl)-propyl-1-phosphonic acid (CPP; Tocris). Patch pipettes were fabricated from borosilicate glass with filament (4–6 MΩ). Intracellular solution contained (in mM): 128 K-gluconate, 10 HEPES, 10 sodium phosphocreatine, 4 MgCl_2_, 4 Na_2_ATP, 3 sodium L-ascorbate, 1 EGTA, and 0.4 Na_2_GTP (pH 7.25; 290 mOsm). To visualize dendritic arbors, 20 µM Alexa 594 or 488 (Molecular Probes) was added to the internal solution. In some cases, 2–3 mg/mL biocytin was included. Electrophysiological signals were amplified with an Axopatch 700B amplifier (Molecular Devices), filtered at 4 KHz, and digitized at 10 KHz. Data I/O were controlled by *Ephus*, a suite of custom Matlab-based (Mathworks) software tools available online (https://openwiki.janelia.org [54]).

Neurons were selected based on pyramidal appearance, or in the case of L4 recordings in vS1, either pyramidal or stellate appearance. In vS1, recordings were generally made in the middle of the barrel field and not a specific whisker barrel. Following patching, a family of current steps was presented to determine firing properties. Neurons with narrow APs and high firing rates were rejected for analysis as presumed interneurons.

### Laser Scanning Photostimulation (LSPS)

Methods followed published procedures [13],[26]. MNI-glutamate (0.2 mM; Tocris, MO [55]) was added to a recirculating bath. Photolysis was performed by shuttering (1.0 ms pulse) the beam of an ultraviolet (355 nm) laser (DPSS Lasers, San Jose, CA), ∼20 mW in the specimen plane, set by a combination of a gradient neutral density filter wheel and Pockels cell (electro-optical modulator; Conoptics). A 16×16 standard stimulus grid for **input maps** had row and column spacing of 110 µm for vM1 and 90 µm for vS1 and S2 recordings. Maps were recorded in voltage clamp at −70 mV. Inhibitory input amplitude was minimized by recording near the chloride reversal potential. The 256 grid sites were visited in a sequence that optimized the spatiotemporal separation between sites [25]. The sequence was repeated 2–4 times per neuron. In vS1 and S2, the map was aligned to the top of the pia and centered on the soma. In vM1, the medial edge of the map was aligned to the interhemispheric fissure, and the top to the dorsal-most edge of pia.

To convert each map's set of traces into an array of pixels that represent response amplitudes, we calculated the average current over a 50 ms post-stimulus window. Direct dendritic responses were excluded on the basis of temporal windowing [56], rejecting traces with events (detected as >3 SD above baseline) with onset latencies of <7 ms. At locations where some maps had direct responses at a given pixel while others did not, the average of the non-direct responses was used; pixels were excluded from the average raw input map for a given neuron if all traces had direct responses.

We measured **excitation profiles** using loose-seal recordings with the amplifier in voltage-follower mode, to gauge the efficacy of photostimulation for neurons in the different layers in the three areas. Excitation profiles were recorded and analyzed following previous methods [13],[25],[26],[30]. To characterize the size of the excitation profile, we calculated the mean weighted distance from the soma of AP generating sites as: Σ(APs × distance from soma)/Σ(APs).

### Connectivity Matrix Analysis

Procedures build on [13]. A transformation step was added, to account for cortical curvature, which was especially strong in vM1. As described in Results, we assigned each point in the stimulus grid a normalized radial distance and horizontal offset (Figure 6). Individual recorded neurons were also assigned a postsynaptic radial distance based on the same criteria. Individual input maps for a given neuron could then be averaged together based on postsynaptic radial distance. Furthermore, when computing the input to a given neuron for the purpose of determining the connectivity matrix, a presynaptic point would be averaged into a bin appropriate to its location. Most aspects of local connectivity were robust to changes in binning. Subsequent corrections to the connectivity matrices were made to account for variations in excitability between layers, and the number of neurons in pre- and post-synaptic layers; these were then presented as neuron→neuron connectivity matrices and layer→layer connectivity matrices (see Results and Text S1).

### Quantitative Comparisons of Connectivity

To make quantitative comparisons between the strength of pathways in different areas, we determined both the average strength of pathways and their variability using a bootstrap-based analysis (Figure 9). After selecting the pre- and postsynaptic neuron populations by relative laminar depth, the strength of corresponding pixels in the input map (limited to maps from neurons in the postsynaptic layer, and pixels in the presynaptic layer within 300 µm horizontal distance of the soma) were averaged for each map. We resampled the individual map averages 10,000 times with replacement and resampled other factors contributing to the individual neuron→neuron strength (number of APs from cortical area's excitation profiles and neuron density). Pathways were presented with the average strength and SD from the bootstrap analysis.

### Analysis of Cortical Lamination

In vS1 and S2, we performed morphometric measurements of cortical landmarks in video images of brain slices. Along a radial line, we marked the locations of the soma, pia, white matter, and major laminar boundaries and calculated the absolute and normalized radial distances of these locations. The bottom extent of cortex was marked at the border between L6 (including the subplate zone) and white matter [57]. The distances to lower borders of layers (±SD) are given in Table 1. The division between L2 and L3 in vS1 was drawn between groups of neurons that did not receive appreciable L4 input (L2 [58]) and those that did. Since this functional division was not clear in S2, L2/3 was divided in half. These values are bracketed in the table. vM1 appearance was similar to somatic motor cortex, with a prominent clear zone in the upper middle part of the cortex, corresponding to L5A [13]. Thus, landmarks indicating the border between L1, a compressed L2/3, and the bottom of L5A were apparent in video images and used to measure laminar boundaries in vM1. The division between L5B and L6 was estimated as the radial distance where cell density increased (Figure S4; Table 1), as a clear border was not apparent based on image contrast. Alternative methods of determining cortical layers in motor and sensory cortex were performed on images of gene expression patterns from the Allen Brain Atlas (Figure S8).

## Supporting Information

Dataset S1 **Connectivity matrix values for neuron- and layer-based connectivity matrices.** Matlab file containing the values for all neuron- and layer-based connectivity matrices of Figure 7. Values are explicitly stated and can be plotted as in the figure.
(0.01 MB TXT)

Figure S1 **Anatomical identification of vS1, vM1, and S2.** (A) Injection of AAV-GFP into vM1 labeled axons projecting to vS1. Left, injection site in medial agranular cortex (vM1). Middle and right, axon termination zones in vS1. Arrow: L4. Str, striatum. cp, cerebral peduncle. VL, ventrolateral nucleus of thalamus. (B) Brainstem targets of vM1 were labeled following AAV-GFP (same animal as above). SpV, spinal trigeminal. 7, facial motor nucleus. (C) Injection of red Lumafluor beads into vM1 labeled somata in vS1. Left, brightfield image of injection site in vM1. Inset, fluorescence image of injection site. Middle, retrograde labeling of vM1-projecting somata in vS1 and S2. Regions of medial agranular cortex (also putative motor regions, as motor cortex is elongated in the anterior/posterior axis) are also labeled. Right, laminar distribution of vM1-projecting neurons in vS1 and S2. Note labeling in L2/3, L5A, and deep in cortical white matter (“L7”). Neurons are less densely labeled in L5B and L6; label is absent in L4. Labeling spreads across multiple barrels. POm, posterior nucleus of thalamus. Agm, medial agranular cortex. Cg, cingulate cortex. (D) Two additional examples of vS1 and S2 labeling following vM1 bead injection. (E) Injection of red Lumafluor beads into vS1 labeled somata in vM1. Left, fluorescent image of injection site in vS1. Right, retrograde labeling of vS1-projecting somata in vM1. VPM, ventroposteromedial nucleus of thalamus. (F) Injection of AAV-GFP into vS1 labeled S2 (left). Injection site is anterior to the plane of the slice. Note the lateral path of axons in white matter and layer 6. Simultaneous injection of red Lumafluor beads retrogradely labeled S2 (right).
(9.85 MB TIF)

Figure S2 **Uncaging MNI-glutamate evokes APs perisomatically.** (A) Cell-attached recording in vM1 with high density (110 µm spacing, top, red) and low density (50 µm spacing, bottom, green) stimulus grids to examine regions of excitation for L5 vM1 neurons. At left, brightfield images with stimulus locations indicated. Middle, traces recorded at each map point. Right, maps quantified to show number and location of evoked APs. Stimulation in L2/3 did not evoke APs in the L5 neurons. (B) Two further examples of cell-attached recordings of L5 vM1 neurons stimulated throughout the width of cortex. (C) Cell-attached recording in L3 of vS1 with two example neurons (top and bottom), presented as above. Uncaging in L4 did not cause spiking of L3 neurons, but perisomatic stimulation did.
(2.95 MB TIF)

Figure S3 **Profile of LSPS photoexcitability and neuron depth in coronal vS1 slices.** (A) Photoexcitability of L3 neurons (blue) measured in loose-seal recording (excitation profiles) as in Figure 2. Total number of APs per map was measured for a soma-centered, 8×8 map with 50 µm spacing and plotted against *z*, depth of soma (slice surface  =  0). Monoexponential fit is shown as a dashed line. (B) Photoexcitability of L5B neurons (red), plotted as above. Monoexponential fit is shown as a dashed line. (C) Photoexcitability of L3 and L5B neurons plotted on the same axes. Note that L5B is more excitable than L3. (D) Photoexcitability of all vS1 neurons (green) plotted with L3 and L5B data. Neurons deeper than 100 µm in the slice did not fire APs.
(0.45 MB TIF)

Figure S4 **Neuronal density in vM1, vS1, and S2.** (A) Coronal sections of mouse brain (50 µm) were stained for the neuronal marker NeuN and red fluorescent secondary antibody. Sections from three animals in vM1 (left), vS1 (middle), and S2 (right) shown. Scale bars, 200 µm. (B) Neuronal somata (red dots) were marked in Neurolucida, and coordinates were imported into Matlab for counting cell numbers as a function of radial distance. An example from vS1 is shown. Pia is at the top of the black rectangle; only neurons within the identified column were counted. (C) Neuron diameter was computed as a function of laminar depth (shown for vS1) and an Abercrombie correction factor [(thickness)/(slice thickness + object diameter)] was used to account for overcounting of neurons at the top and bottom of the section. (D) Neuronal density as a function of laminar position is plotted for vM1 (blue), vS1 (red), and S2 (green). Thick lines indicate the average of three sections. Thin lines indicate density for individual sections. Laminar boundaries for vS1 are indicated as horizontal lines.
(2.03 MB TIF)

Figure S5 **Examples of vM1 input maps.** (A) Bright-field image of vM1, with overlaid LSPS grid (16×16 sites, 110 µm spacing), aligned to pia medially and superiorly. (B) Example traces from three neurons' maps (boxed regions in C). Circles: somata. Dendritic responses omitted. (C) Examples of input maps for neurons at different laminar depths. Three examples are given (in a column) for each radial distance from the pia. Grouping corresponds to Figure 3. Normalized distances and approximate layers are indicated above the maps. White boxes indicate regions enlarged in (B). At right, schematic showing the relative locations of L2/3, L5A, L5B, and L6 for reference. Black pixels: dendritic response sites. Circles: somata. Color scale applies to all maps.
(0.55 MB TIF)

Figure S6 **Examples of vS1 input maps.** (A) Bright-field image of vS1, with overlaid LSPS grid (16×16 sites, 90 µm spacing), aligned to pia superiorly and over neuron horizontally. (B) Example traces from three neurons' maps (boxed regions in C). Circles: somata. Dendritic responses omitted. (C) Examples of input maps for neurons at different laminar depths. Three examples are given (in a column) for each radial distance from the pia. Grouping corresponds to Figure 4. Normalized distances and layers are indicated above the maps. White boxes indicate regions enlarged in (B). On rightmost maps, markers are given to indicate position of laminar boundaries between cortical layers. Black pixels: dendritic response sites. Circles: somata. Color scale applies to all maps.
(0.55 MB TIF)

Figure S7 **Examples of S2 input maps.** (A) Bright-field image of S2 at left (lateral) end of vS1, with overlaid LSPS grid (16×16 sites, 90 µm spacing), aligned to pia superiorly and over neuron horizontally. S2 maps are aligned such that the medial side (S1) is to the left, as indicated below the image. (B) Example traces from three neurons' maps (boxed regions in C). Circles: somata. Dendritic responses omitted. (C) Examples of input maps for neurons at different laminar depths. Three examples are given (in a column) for each radial distance from the pia. Grouping corresponds to Figure 5. Normalized distances and approximate layers are indicated above the maps. White boxes indicate regions enlarged in (B). On rightmost maps, markers are given to indicate position of laminar boundaries between cortical layers. Black pixels: dendritic response sites. Circles: somata. Color scale applies to all maps.
(0.57 MB TIF)

Figure S8 **Comparison of molecular and anatomical definition of cortical lamination.** (A, B) Sample in situ images of gene expression from coronal sections of vM1 in the Allen Brain Atlas. Etv1 and Wfs1 illustrated. (C) Brightfield image of vM1 with the axis along which measurements of relative laminar depth were taken indicated. Cortex is marked from 0 (pia) to 1 (white matter). White marks to the right of the ladder indicate cytoarchitectonic boundaries based on the video image. (D) Molecularly defined layers for vM1 plotted for comparison. Radial distance at which Etv1, Wfs1, Rorβ, Plexin D1, Enc1, Abat, and Fezf2 expression were measured based on images in the Allen Brain atlas. Boundaries indicate onset and offset of expression. There were two laminae for PlexinD1 and Enc1. Etv1 expression contained bands of high (superficial) and low (deep) expression. Thy1-ChR2 mouse (line 18) expressed in L5 neurons in motor cortex. Summary table of measurements for vM1 and vS1 are given below.
(3.36 MB PDF)

Figure S9 **Analysis of horizontal and oblique pathways.** (A) Each vM1 neuron's input map was projected onto a vector representing the horizontal profile of synaptic input. The vectors were sorted by postsynaptic position. (B) Same data as for (A), but grouped into distance bins and averaged. Equivalent to projection of the 3-D map data array onto the postsynaptic-horizontal plane. (C) Projection of the 3-D map data array onto the presynaptic-horizontal plane (orthogonal to B). (D–F) Same analyses as (A–C), for vS1 data set. (G–I) Same analyses as (A–C), for S2 data set. (J–L) Horizontal-only analysis. Only “home-layer” (intralaminar; ±0.1 radial distance) data were used to generate each neuron's horizontal vector. For example, top vectors show L2 horizontal inputs to L2. Left: all neurons' vectors, sorted by postsynaptic position. Middle: vectors were grouped into distance-based bins and averaged. Black pixels: sites <100 µm from soma were excluded, and the top bin (corresponding to L1) was empty. Right: Mean weighted distance of individual and averaged horizontal data as a function of postsynaptic position. Values represent the mean, for each neuron, of the vector values times the pixel distances from the soma. Lines with error bars represent mean ± s.e.m.
(0.66 MB TIF)

Figure S10 **Construction of neuron→neuron connectivity matrices.** (A) Example of how neuron→neuron connectivity matrices are constructed; vS1 is used for this example. Given the individual input vectors to a given neuron (Figure 6A–G) averaged in evenly spaced bins, these data are presented for all neurons as an “uncaging→neuron” matrix (left). Cell-type specific excitability is accounted for by dividing each presynaptic bin by the average number of AP per region (top). Corrections are shown as a 2D matrix; corrections are the same for all columns along the presynaptic orientation. Furthermore, input is divided by presynaptic cell density to correct for the number of neurons activated per uncaging event (bottom). Thus, a neuron→neuron connectivity matrix is presented (right). (B) Data presented as in (A), but with postsynaptic neurons binned. Average laminar borders are superimposed. Connectivity matrices in this style are presented in Figures 6 and 7.
(0.45 MB TIF)

Figure S11 **Construction of neuron→neuron connectivity matrices in cortical layer bins.** (A) Example of how neuron→neuron connectivity matrices are constructed; vS1 is used for this example. Process is identical to Figure S10, but bins are determined based on boundaries between cortical layers instead of even spacing. Given the individual input vectors to a given neuron (Figure 6A) averaged in laminar specific bins, these data are presented for all neurons as an uncaging→neuron matrix (left). Cell-type specific excitability was accounted for by dividing each presynaptic bin by the average number of AP per region. Corrections are shown as a 2D matrix; corrections are the same for all columns along the presynaptic orientation. Furthermore, input was divided by presynaptic cell density to correct for the number of neurons activated per uncaging event, resulting in a neuron→neuron connectivity matrix (right). (B) Data presented as in (A), but with postsynaptic neurons binned into cortical layer specific bins.
(0.39 MB TIF)

Figure S12 **Construction of layer→layer connectivity matrices in cortical layer bins.** (A) Example of how layer→layer connectivity matrices are constructed from neuron→neuron connectivity matrices; vS1 is used for this example. The corrected neuron→neuron matrix of Figure S11B (right) is used as a starting point. For the purpose of determining total number of neurons per layer, instead of density, a cortical column of 300×300 µm was used in the plane normal to the radial axis from pia to white matter. The thickness of each layer along the radial axis was based on cytoarchitectonic measurements (Table 1); density was based on Figure S4. Correction to the neuron→neuron matrix involved multiplication by both the number of presynaptic (bottom; columns) and postsynaptic (top; rows) neurons. Connectivity matrices in this style are presented in Figure 7.
(0.23 MB TIF)

Figure S13 **Quantitative comparison of neuron→neuron connectivity derived from complementary methods.** (A) Matrices of neuron→neuron connectivity based on pair recordings [27] and LSPS (Hooks et al., this work) plotted on the same scale. Single cell connectivity for pair recordings is converted from peak amplitude in mV to pA using layer specific input resistance, and multiplied by connection probability. Single cell connectivity for LSPS is converted from mean amplitude in pA to peak amplitude using a conversion factor of 0.2 (based on ratio of mean/peak amplitude in LSPS recordings).
(0.43 MB TIF)

Figure S14 **Functional motor mapping using optical microstimulation.** (A) Coronal brain slice through motor cortex, prepared from a Thy1-ChR2-YFP (line 18) mouse (scale bar: 0.5 mm). (B) Functional motor map of whisker (red), forelimb (green), and tongue (blue), aligned to bregma (Br) and averaged across six animals. Distances are in mm from the midline or bregma. Colored lines delimit the area where movement could be evoked at threshold power in at least 50% of trials. The grid of the stimulation pattern is indicated with light blue dots (0.5 mm spacing, scale in mm).
(2.18 MB TIF)

Figure S15 **Channelrhodopsin-based mapping of the extent of axonal innervations of local circuits in brain slice.** (A) vM1 neurons were transfected with channelrhodopsin-2 by stereotactic injection of adeno-associated virus. vM1 slices were prepared as for LSPS circuit mapping and placed in normal artificial cerebrospinal fluid containing 2 mM Ca^2+^ and 1 mM Mg^2+^, as well as 5 µM CPP and 10 µM NBQX to block excitatory synaptic transmission. L2/3 pyramidal neurons were then recorded in cell-attached configuration while exciting the slice with 1 ms pulses of 1 mW 473 nm laser light. Grid spacing was selected to cover a large area (110×110 µm, 16×16) or a detailed area (50×50 µm, 12×26). Points where an action potential was evoked are indicated in red overlay; points without excitation are shown in gray. White circle indicates L2/3 soma. Left panels are large area maps overlaid on the slice image. The right image in (A) demonstrates the spatial resolution (∼50 µm) of the excitation on a medial extending branch of the axon. (B, C, D) Additional examples presented in the same format.
(2.80 MB TIF)

Figure S16 **Input mapping of afferents to L5A neurons in vibrissal motor cortex using opposite faces of adjacent brain slices.** (A) Experimental design. Two adjacent brain slices were prepared from the same animal. The posterior side of the anterior slice (PSAS) and anterior side of the posterior slice (ASPS) were used for recording (as previously described). (B) Representative input maps from neurons on PSAS (top) and ASPS (bottom) slices. (C) Group data for *n*  =  11 neurons on each side. Average input strength is quantified as the mean of the input vector for each given presynaptic depth bin, and presented ± s. e. m.
(0.49 MB TIF)

Table S1 **Connectivity matrix values for neuron- and layer-based connectivity matrices.** Excel file containing the values for all neuron- and layer-based connectivity matrices of Figure 7.
(0.03 MB XLS)

Text S1 **Laminar analysis of excitatory local circuits in vibrissal motor and sensory cortical areas.** Supplemental information to the article. Sections include: Supplemental Methods (relationship of pixel values in input maps to average synaptic connection strength (*q*_con_); converting input maps to connectivity matrices; methods for optical microstimulation), Supplemental Discussion (comparison and limitations of circuit mapping techniques; horizontal connectivity), and References.
(0.32 MB RTF)

## Abbreviations

APs: action potentials
ChR2: channelrhodopsin-2
LSPS: laser scanning photostimulation

## References

[1] Ahissar E (2008). And motion changes it all.. Nat Neurosci.

[2] Kleinfeld D, Ahissar E, Diamond M. E (2006). Active sensation: insights from the rodent vibrissa sensorimotor system.. Curr Opin Neurobiol.

[3] Ahissar E, Arieli A (2001). Figuring space by time.. Neuron.

[4] Gibson J. J (1962). Observations on active touch.. Psychol Rev.

[5] Nelson M. E, MacIver M. A (2006). Sensory acquisition in active sensing systems.. J Comp Physiol A Neuroethol Sens Neural Behav Physiol.

[6] Nguyen Q. T, Kleinfeld D (2005). Positive feedback in a brainstem tactile sensorimotor loop.. Neuron.

[7] Urbain N, Deschenes M (2007). Motor cortex gates vibrissal responses in a thalamocortical projection pathway.. Neuron.

[8] Chakrabarti S, Alloway K. D (2006). Differential origin of projections from SI barrel cortex to the whisker representations in SII and MI.. J Comp Neurol.

[9] Armstrong-James M, Fox K (1987). Spatiotemporal convergence and divergence in the rat S1 “barrel” cortex.. J Comp Neurol.

[10] Ferezou I, Haiss F, Gentet L. J, Aronoff R, Weber B (2007). Spatiotemporal dynamics of cortical sensorimotor integration in behaving mice.. Neuron.

[11] Hoffer Z. S, Hoover J. E, Alloway K. D (2003). Sensorimotor corticocortical projections from rat barrel cortex have an anisotropic organization that facilitates integration of inputs from whiskers in the same row.. J Comp Neurol.

[12] Zilles K, Amunts K (2010). Centenary of Brodmann's map—conception and fate.. Nat Rev Neurosci.

[13] Weiler N, Wood L, Yu J, Solla S. A, Shepherd G. M (2008). Top-down laminar organization of the excitatory network in motor cortex.. Nat Neurosci.

[14] Anderson C. T, Sheets P. L, Kiritani T, Shepherd G. M (2010). Sublayer-specific microcircuits of corticospinal and corticostriatal neurons in motor cortex.. Nat Neurosci.

[15] Brecht M, Krauss A, Muhammad S, Sinai-Esfahani L, Bellanca S (2004). Organization of rat vibrissa motor cortex and adjacent areas according to cytoarchitectonics, microstimulation, and intracellular stimulation of identified cells.. J Comp Neurol.

[16] Caviness V. S (1975). Architectonic map of neocortex of the normal mouse.. J Comp Neurol.

[17] Li C. X, Waters R. S (1991). Organization of the mouse motor cortex studied by retrograde tracing and intracortical microstimulation (ICMS) mapping.. Can J Neurol Sci.

[18] Grinevich V, Brecht M, Osten P (2005). Monosynaptic pathway from rat vibrissa motor cortex to facial motor neurons revealed by lentivirus-based axonal tracing.. J Neurosci.

[19] Ayling O. G, Harrison T. C, Boyd J. D, Goroshkov A, Murphy T. H (2009). Automated light-based mapping of motor cortex by photoactivation of channelrhodopsin-2 transgenic mice.. Nat Methods.

[20] Hira R, Honkura N, Noguchi J, Maruyama Y, Augustine G. J (2009). Transcranial optogenetic stimulation for functional mapping of the motor cortex.. J Neurosci Methods.

[21] Woolsey T. A, Van der Loos H (1970). The structural organization of layer IV in the somatosensory region (SI) of mouse cerebral cortex. The description of a cortical field composed of discrete cytoarchitectonic units.. Brain Res.

[22] Carvell G. E, Simons D. J (1986). Somatotopic organization of the second somatosensory area (SII) in the cerebral cortex of the mouse.. Somatosens Res.

[23] Callaway E. M, Katz L. C (1993). Photostimulation using caged glutamate reveals functional circuitry in living brain slices.. Proc Natl Acad Sci U S A.

[24] Katz L. C, Dalva M. B (1994). Scanning laser photostimulation: a new approach for analyzing brain circuits.. J Neurosci Methods.

[25] Shepherd G. M, Pologruto T. A, Svoboda K (2003). Circuit analysis of experience-dependent plasticity in the developing rat barrel cortex.. Neuron.

[26] Shepherd G. M. G, Svoboda K (2005). Laminar and columnar organization of ascending excitatory projections to layer 2/3 pyramidal neurons in rat barrel cortex.. J Neurosci.

[27] Lefort S, Tomm C, Floyd Sarria J. C, Petersen C. C (2009). The excitatory neuronal network of the C2 barrel column in mouse primary somatosensory cortex.. Neuron.

[28] von Economo C (1929). The cytoarchitectonics of the human cerebral cortex..

[29] Schubert D, Kotter R, Zilles K, Luhmann H. J, Staiger J. F (2003). Cell type-specific circuits of cortical layer IV spiny neurons.. J Neurosci.

[30] Bureau I, Shepherd G. M, Svoboda K (2004). Precise development of functional and anatomical columns in the neocortex.. Neuron.

[31] Stepanyants A, Hirsch J. A, Martinez L. M, Kisvarday, ZF, Ferecsko, AS, Chklovskii, DB (2008). Local potential connectivity in cat primary visual cortex.. Cereb Cortex.

[32] Binzegger T, Douglas R. J, Martin K. A (2004). A quantitative map of the circuit of cat primary visual cortex.. J Neurosci.

[33] Song S, Sjostrom P. J, Reigl M, Nelson S, Chklovskii D. B (2005). Highly nonrandom features of synaptic connectivity in local cortical circuits.. PLoS Biol.

[34] Holmgren C, Harkany T, Svennenfors B, Zilberter Y (2003). Pyramidal cell communication within local networks in layer 2/3 of rat neocortex.. J Physiol.

[35] Morishima M, Kawaguchi Y (2006). Recurrent connection patterns of corticostriatal pyramidal cells in frontal cortex.. J Neurosci.

[36] Shepherd G. M, Stepanyants A, Bureau I, Chklovskii D, Svoboda K (2005). Geometric and functional organization of cortical circuits.. Nat Neurosci.

[37] Yu J, Anderson C. T, Kiritani T, Sheets P. L, Wokosin D. L (2008). Local-circuit phenotypes of layer 5 neurons in motor-frontal cortex of YFP-H mice.. Front Neural Circuits.

[38] Schuz A, Palm G (1989). Density of neurons and synapses in the cerebral cortex of the mouse.. J Comp Neurol.

[39] Tsai P. S, Kaufhold J. P, Blinder P, Friedman B, Drew P. J (2009). Correlations of neuronal and microvascular densities in murine cortex revealed by direct counting and colocalization of nuclei and vessels.. J Neurosci.

[40] Kampa B. M, Letzkus J. J, Stuart G. J (2006). Cortical feed-forward networks for binding different streams of sensory information.. Nat Neurosci.

[41] Yoshimura Y, Dantzker J. L, Callaway E. M (2005). Excitatory cortical neurons form fine-scale functional networks.. Nature.

[42] Petreanu L, Mao T, Sternson S. M, Svoboda K (2009). The subcellular organization of neocortical excitatory connections.. Nature.

[43] Brown S. P, Hestrin S (2009). Intracortical circuits of pyramidal neurons reflect their long-range axonal targets.. Nature.

[44] Dantzker J. L, Callaway E. M (2000). Laminar sources of synaptic input to cortical inhibitory interneurons and pyramidal neurons.. Nat Neurosci.

[45] Barbour D. L, Callaway E. M (2008). Excitatory local connections of superficial neurons in rat auditory cortex.. J Neurosci.

[46] Oviedo H. V, Bureau I, Svoboda K, Zador A. M (2010). The functional asymmetry of auditory cortex is reflected in the organization of local cortical circuits.. Nat Neurosci 13:.

[47] Tarczy-Hornoch K, Martin K. A, Stratford K. J, Jack J. J (1999). Intracortical excitation of spiny neurons in layer 4 of cat striate cortex in vitro.. Cereb Cortex.

[48] Lee C. C, Sherman S. M (2008). Synaptic properties of thalamic and intracortical inputs to layer 4 of the first- and higher-order cortical areas in the auditory and somatosensory systems.. J Neurophysiol.

[49] Andjelic S, Gallopin T, Cauli B, Hill E. L, Roux L (2009). Glutamatergic nonpyramidal neurons from neocortical layer VI and their comparison with pyramidal and spiny stellate neurons.. J Neurophysiol.

[50] Petreanu L, Huber D, Sobczyk A, Svoboda K (2007). Channelrhodopsin-2-assisted circuit mapping of long-range callosal projections.. Nat Neurosci.

[51] Sirota M. G, Swadlow H. A, Beloozerova I. N (2005). Three channels of corticothalamic communication during locomotion.. J Neurosci.

[52] Zarrinpar A, Callaway E. M (2006). Local connections to specific types of layer 6 neurons in the rat visual cortex.. J Neurophysiol.

[53] Svoboda K, Hooks B. M, Shepherd G. M. G (2010). Barrel Cortex.. Handbook of Brain Microcircuits.

[54] Suter B. A, O'Connor T, Iyer V, Petreanu L. T, Hooks B. M, Kiritani T, Svoboda K, Shepherd G. M. G (2010). Ephus: multipurpose data acquisition software for neuroscience experiments.. Front. Neurosci.

[55] Canepari M, Nelson L, Papageorgiou G, Corrie J. E, Ogden D (2001). Photochemical and pharmacological evaluation of 7-nitroindolinyl-and 4-methoxy-7-nitroindolinyl-amino acids as novel, fast caged neurotransmitters.. J Neurosci Methods.

[56] Schubert D, Staiger J. F, Cho N, Kotter R, Zilles K (2001). Layer-specific intracolumnar and transcolumnar functional connectivity of layer V pyramidal cells in rat barrel cortex.. J Neurosci.

[57] Torres-Reveron J, Friedlander M. J (2007). Properties of persistent postnatal cortical subplate neurons.. J Neurosci.

[58] Bureau I, von Saint Paul F, Svoboda K (2006). Interdigitated paralemniscal and lemniscal pathways in the mouse barrel cortex.. PLoS Biol.

