# Supplementary figures and images for: Laminar Analysis of Excitatory Local Circuits in Vibrissal Motor and Sensory Cortical Areas
Source: PLoS Biol. 2011 Jan 4;9(1):e1000572. doi: 10.1371/journal.pbio.1000572 (PMC3014926; doi:10.1371/journal.pbio.1000572)

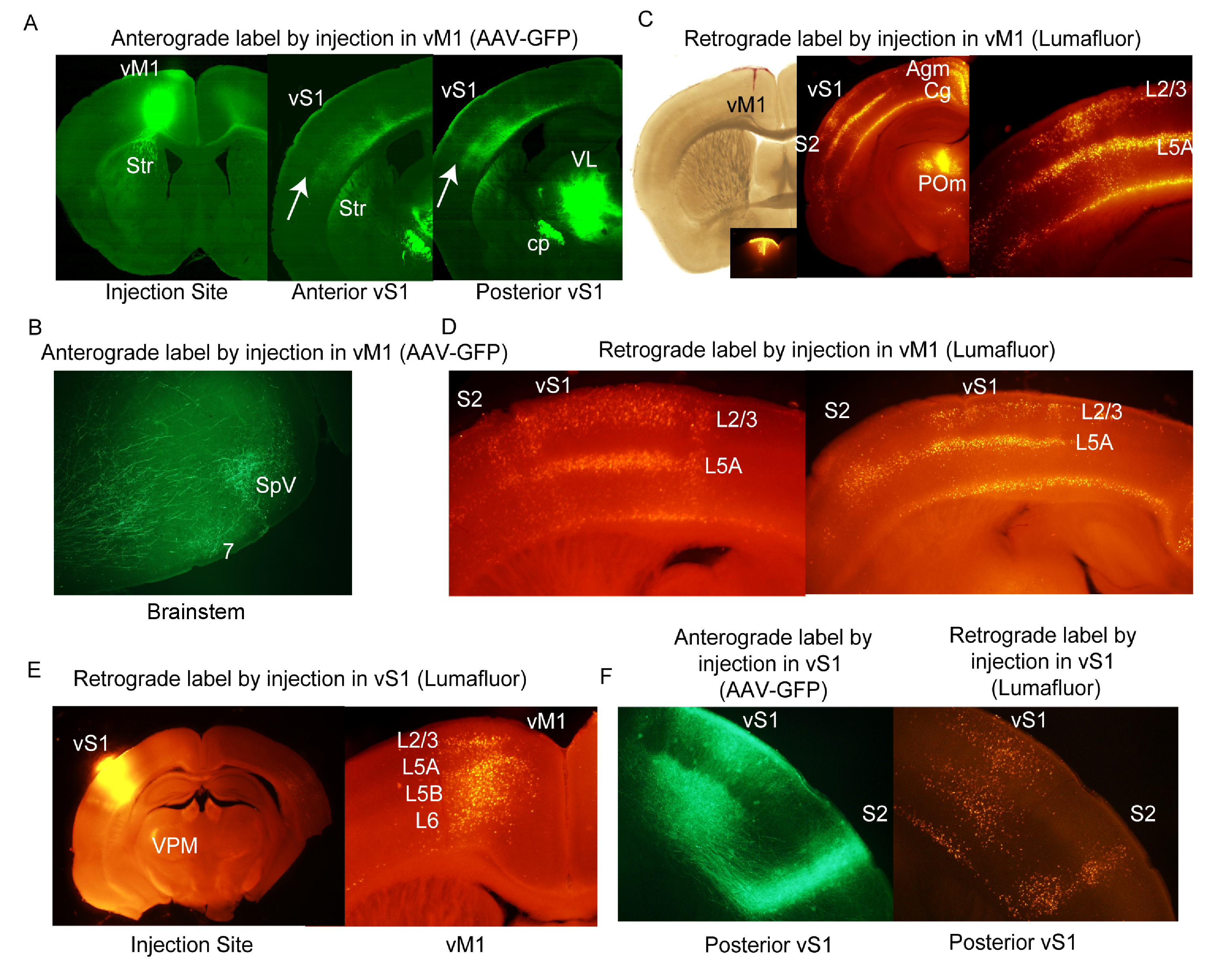

Supplement: Figure S1 — Anatomical identification of vS1, vM1, and S2. (A) Injection of AAV-GFP into vM1 labeled axons projecting to vS1. Left, injection site in medial agranular cortex (vM1). Middle and right, axon termination zones in vS1. Arrow: L4. Str, striatum. cp, cerebral peduncle. VL, ventrolateral nucleus of thalamus. (B) Brainstem targets of vM1 were labeled following AAV-GFP (same animal as above). SpV, spinal trigeminal. 7, facial motor nucleus. (C) Injection of red Lumafluor beads into vM1 labeled somata in vS1. Left, brightfield image of injection site in vM1. Inset, fluorescence image of injection site. Middle, retrograde labeling of vM1-projecting somata in vS1 and S2. Regions of medial agranular cortex (also putative motor regions, as motor cortex is elongated in the anterior/posterior axis) are also labeled. Right, laminar distribution of vM1-projecting neurons in vS1 and S2. Note labeling in L2/3, L5A, and deep in cortical white matter (“L7”). Neurons are less densely labeled in L5B and L6; label is absent in L4. Labeling spreads across multiple barrels. POm, posterior nucleus of thalamus. Agm, medial agranular cortex. Cg, cingulate cortex. (D) Two additional examples of vS1 and S2 labeling following vM1 bead injection. (E) Injection of red Lumafluor beads into vS1 labeled somata in vM1. Left, fluorescent image of injection site in vS1. Right, retrograde labeling of vS1-projecting somata in vM1. VPM, ventroposteromedial nucleus of thalamus. (F) Injection of AAV-GFP into vS1 labeled S2 (left). Injection site is anterior to the plane of the slice. Note the lateral path of axons in white matter and layer 6. Simultaneous injection of red Lumafluor beads retrogradely labeled S2 (right). (9.85 MB TIF) [file pbio.1000572.s002.tif]

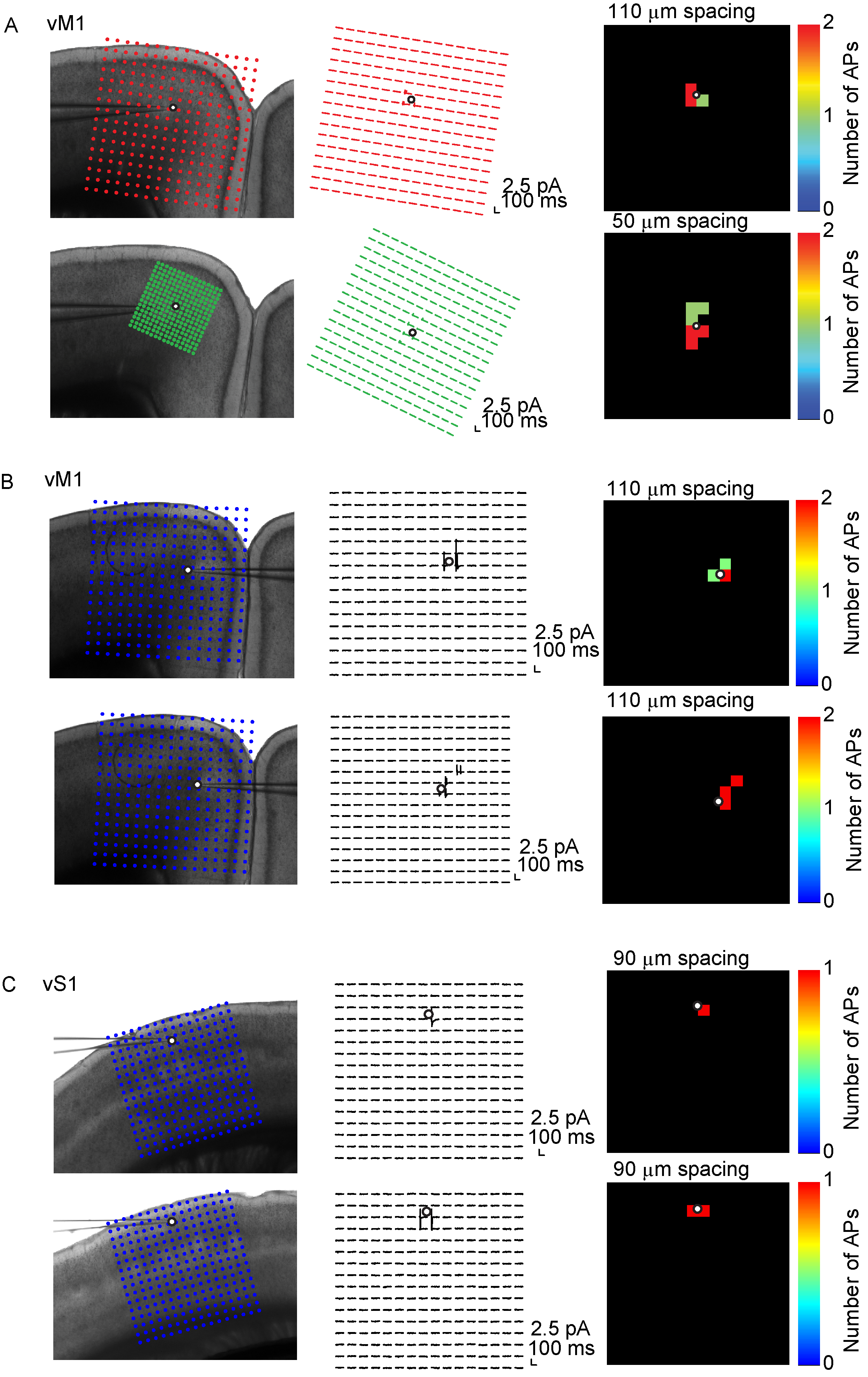

Supplement: Figure S2 — Uncaging MNI-glutamate evokes APs perisomatically. (A) Cell-attached recording in vM1 with high density (110 µm spacing, top, red) and low density (50 µm spacing, bottom, green) stimulus grids to examine regions of excitation for L5 vM1 neurons. At left, brightfield images with stimulus locations indicated. Middle, traces recorded at each map point. Right, maps quantified to show number and location of evoked APs. Stimulation in L2/3 did not evoke APs in the L5 neurons. (B) Two further examples of cell-attached recordings of L5 vM1 neurons stimulated throughout the width of cortex. (C) Cell-attached recording in L3 of vS1 with two example neurons (top and bottom), presented as above. Uncaging in L4 did not cause spiking of L3 neurons, but perisomatic stimulation did. (2.95 MB TIF) [file pbio.1000572.s003.tif]

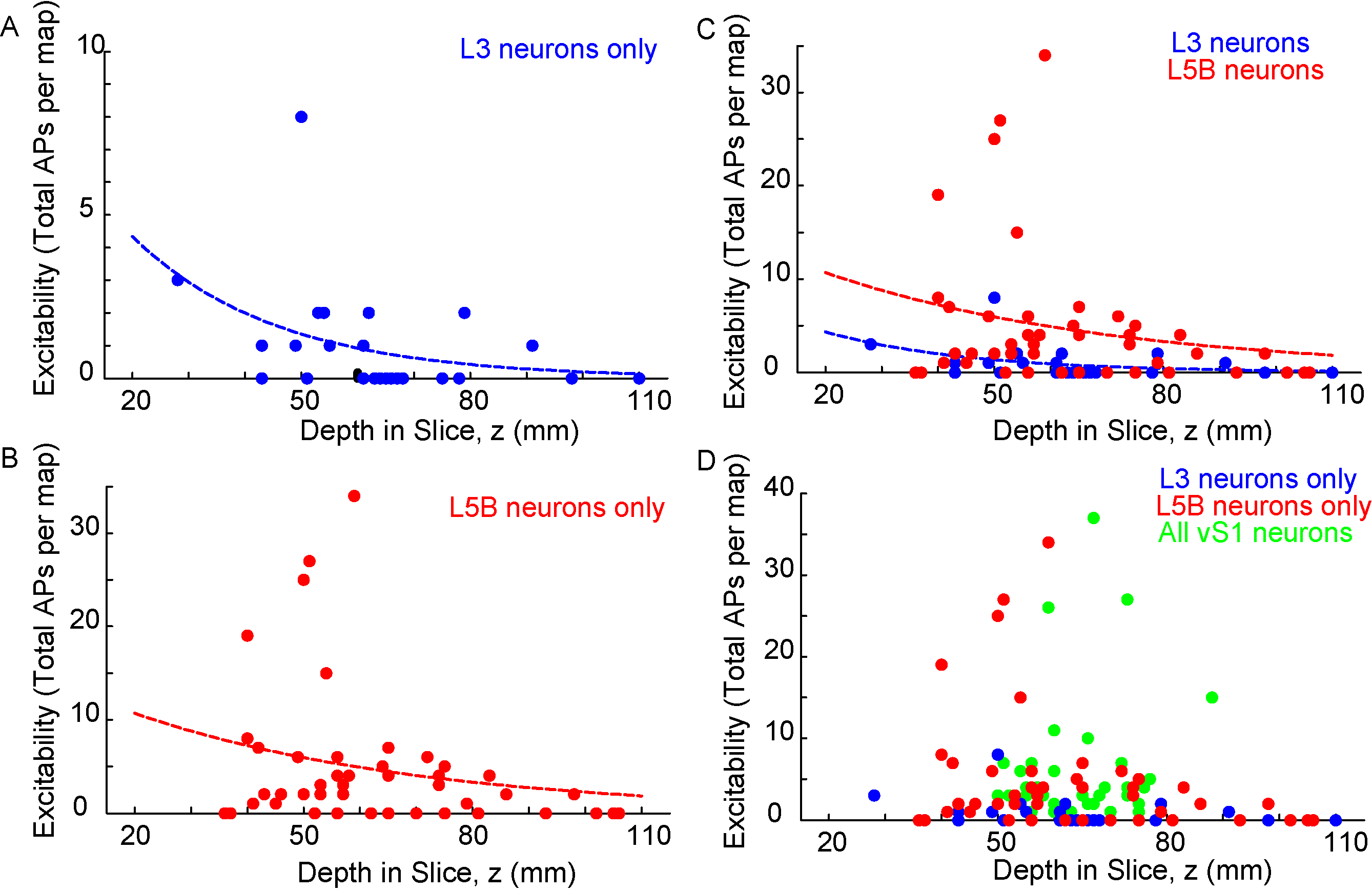

Supplement: Figure S3 — Profile of LSPS photoexcitability and neuron depth in coronal vS1 slices. (A) Photoexcitability of L3 neurons (blue) measured in loose-seal recording (excitation profiles) as in Figure 2. Total number of APs per map was measured for a soma-centered, 8×8 map with 50 µm spacing and plotted against z, depth of soma (slice surface = 0). Monoexponential fit is shown as a dashed line. (B) Photoexcitability of L5B neurons (red), plotted as above. Monoexponential fit is shown as a dashed line. (C) Photoexcitability of L3 and L5B neurons plotted on the same axes. Note that L5B is more excitable than L3. (D) Photoexcitability of all vS1 neurons (green) plotted with L3 and L5B data. Neurons deeper than 100 µm in the slice did not fire APs. (0.45 MB TIF) [file pbio.1000572.s004.tif]

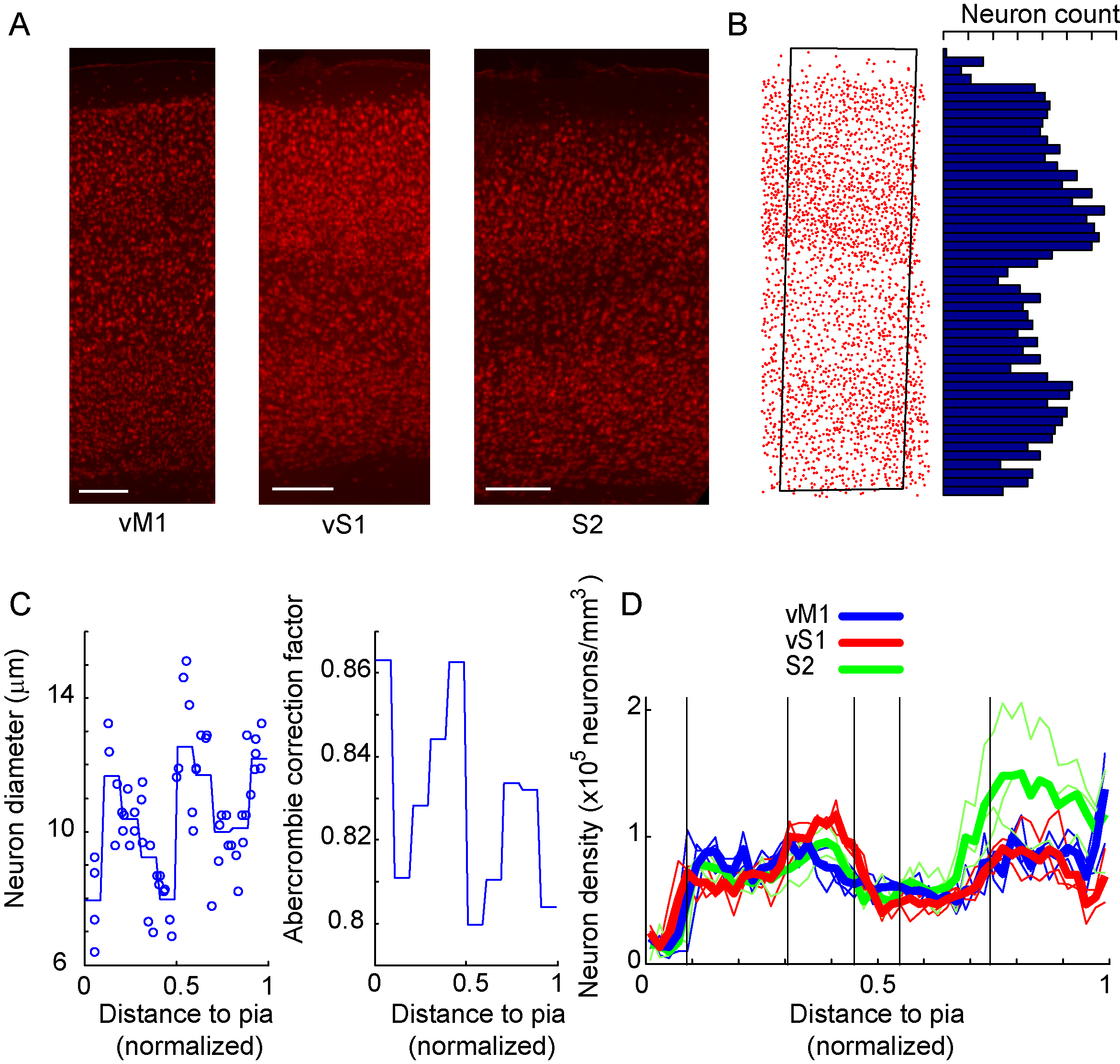

Supplement: Figure S4 — Neuronal density in vM1, vS1, and S2. (A) Coronal sections of mouse brain (50 µm) were stained for the neuronal marker NeuN and red fluorescent secondary antibody. Sections from three animals in vM1 (left), vS1 (middle), and S2 (right) shown. Scale bars, 200 µm. (B) Neuronal somata (red dots) were marked in Neurolucida, and coordinates were imported into Matlab for counting cell numbers as a function of radial distance. An example from vS1 is shown. Pia is at the top of the black rectangle; only neurons within the identified column were counted. (C) Neuron diameter was computed as a function of laminar depth (shown for vS1) and an Abercrombie correction factor [(thickness)/(slice thickness + object diameter)] was used to account for overcounting of neurons at the top and bottom of the section. (D) Neuronal density as a function of laminar position is plotted for vM1 (blue), vS1 (red), and S2 (green). Thick lines indicate the average of three sections. Thin lines indicate density for individual sections. Laminar boundaries for vS1 are indicated as horizontal lines. (2.03 MB TIF) [file pbio.1000572.s005.tif]

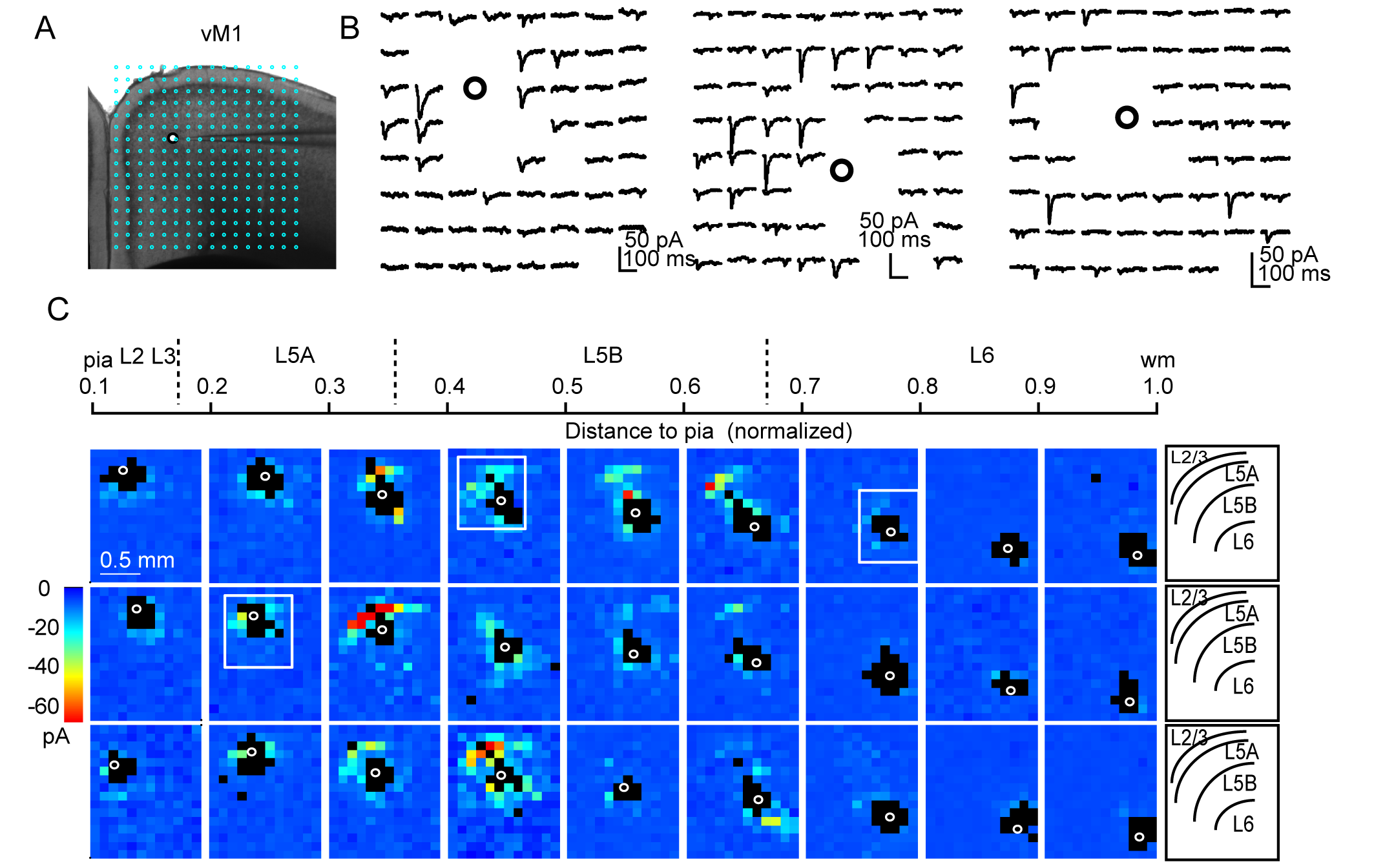

Supplement: Figure S5 — Examples of vM1 input maps. (A) Bright-field image of vM1, with overlaid LSPS grid (16×16 sites, 110 µm spacing), aligned to pia medially and superiorly. (B) Example traces from three neurons' maps (boxed regions in C). Circles: somata. Dendritic responses omitted. (C) Examples of input maps for neurons at different laminar depths. Three examples are given (in a column) for each radial distance from the pia. Grouping corresponds to Figure 3. Normalized distances and approximate layers are indicated above the maps. White boxes indicate regions enlarged in (B). At right, schematic showing the relative locations of L2/3, L5A, L5B, and L6 for reference. Black pixels: dendritic response sites. Circles: somata. Color scale applies to all maps. (0.55 MB TIF) [file pbio.1000572.s006.tif]

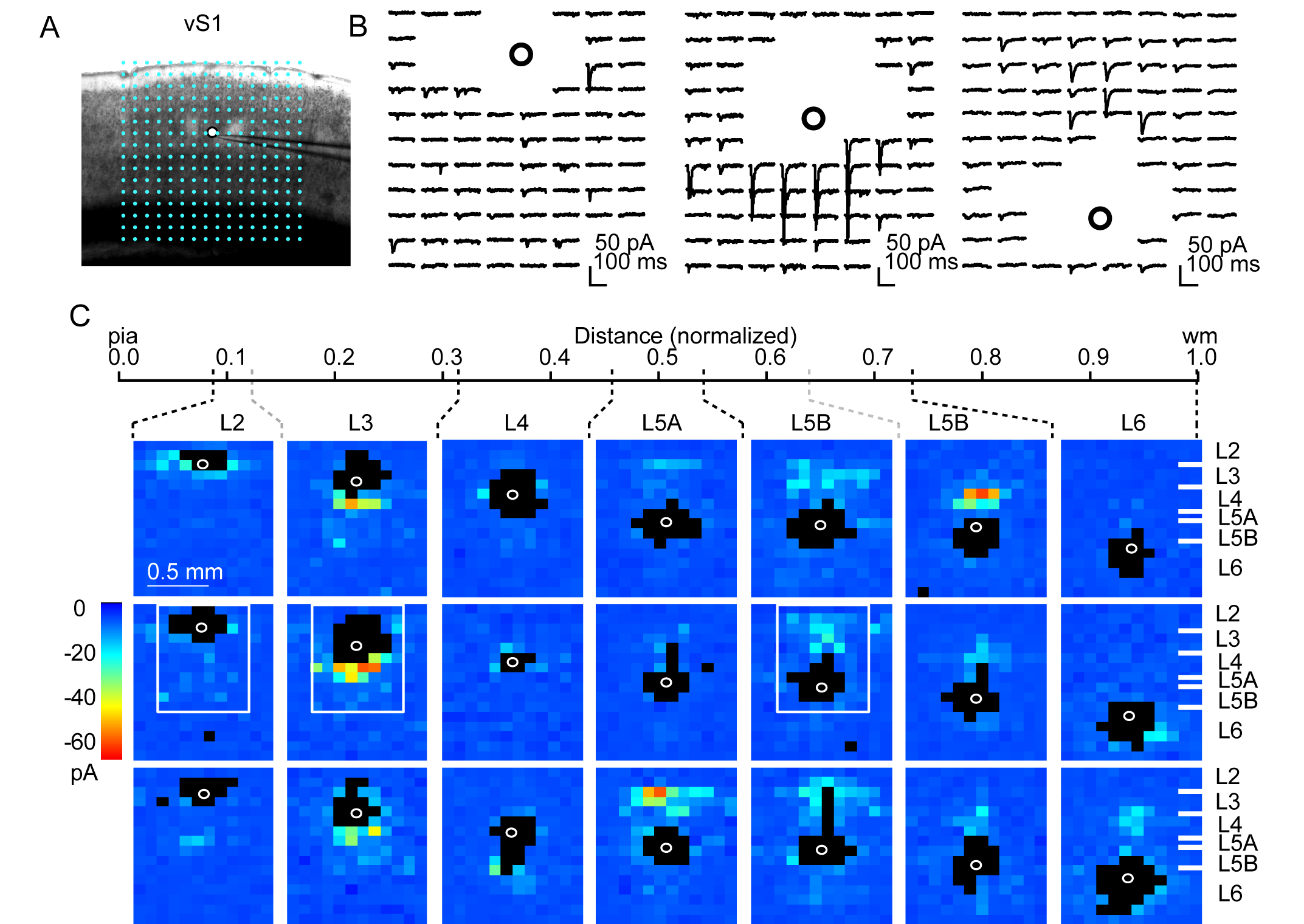

Supplement: Figure S6 — Examples of vS1 input maps. (A) Bright-field image of vS1, with overlaid LSPS grid (16×16 sites, 90 µm spacing), aligned to pia superiorly and over neuron horizontally. (B) Example traces from three neurons' maps (boxed regions in C). Circles: somata. Dendritic responses omitted. (C) Examples of input maps for neurons at different laminar depths. Three examples are given (in a column) for each radial distance from the pia. Grouping corresponds to Figure 4. Normalized distances and layers are indicated above the maps. White boxes indicate regions enlarged in (B). On rightmost maps, markers are given to indicate position of laminar boundaries between cortical layers. Black pixels: dendritic response sites. Circles: somata. Color scale applies to all maps. (0.55 MB TIF) [file pbio.1000572.s007.tif]

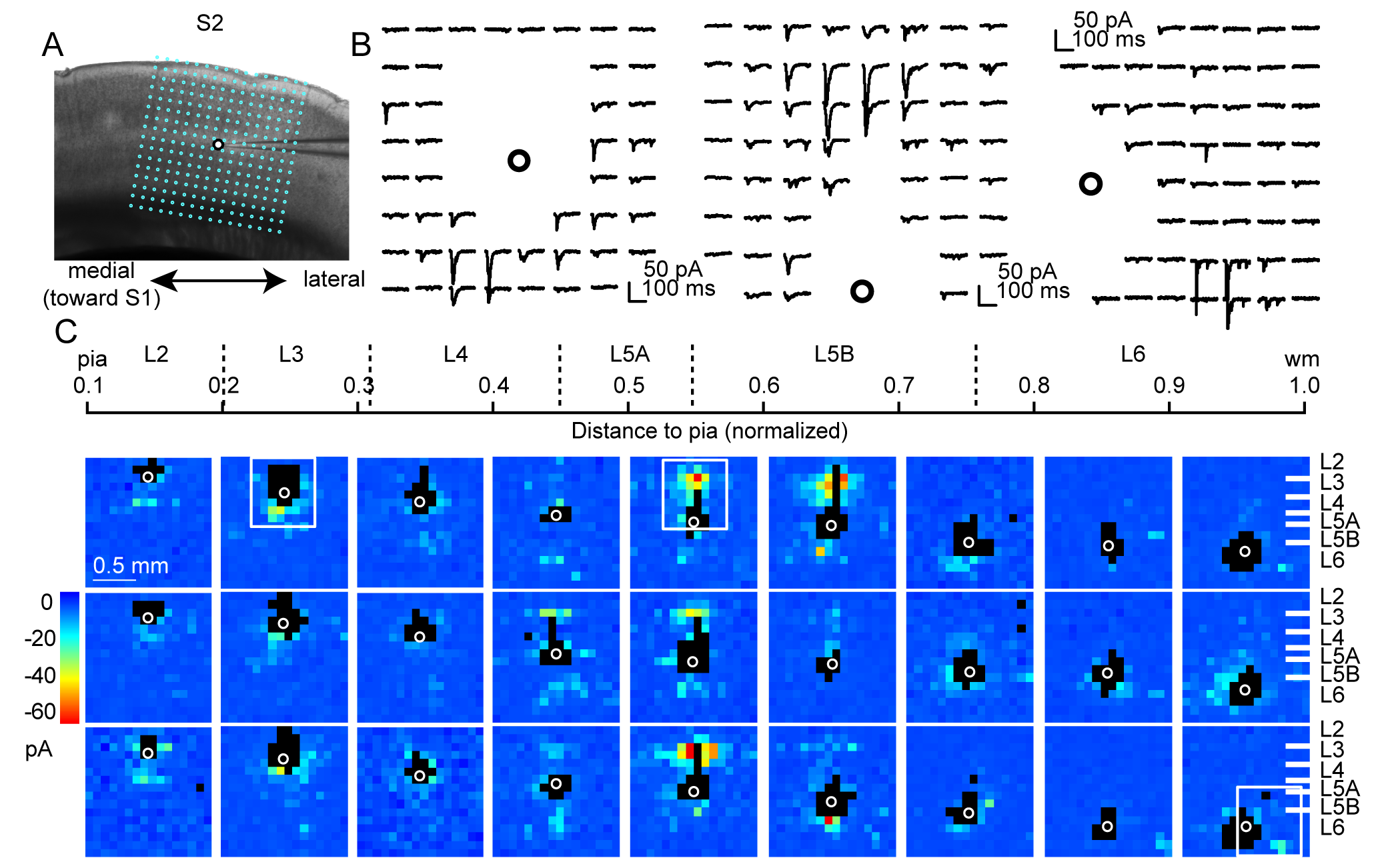

Supplement: Figure S7 — Examples of S2 input maps. (A) Bright-field image of S2 at left (lateral) end of vS1, with overlaid LSPS grid (16×16 sites, 90 µm spacing), aligned to pia superiorly and over neuron horizontally. S2 maps are aligned such that the medial side (S1) is to the left, as indicated below the image. (B) Example traces from three neurons' maps (boxed regions in C). Circles: somata. Dendritic responses omitted. (C) Examples of input maps for neurons at different laminar depths. Three examples are given (in a column) for each radial distance from the pia. Grouping corresponds to Figure 5. Normalized distances and approximate layers are indicated above the maps. White boxes indicate regions enlarged in (B). On rightmost maps, markers are given to indicate position of laminar boundaries between cortical layers. Black pixels: dendritic response sites. Circles: somata. Color scale applies to all maps. (0.57 MB TIF) [file pbio.1000572.s008.tif]

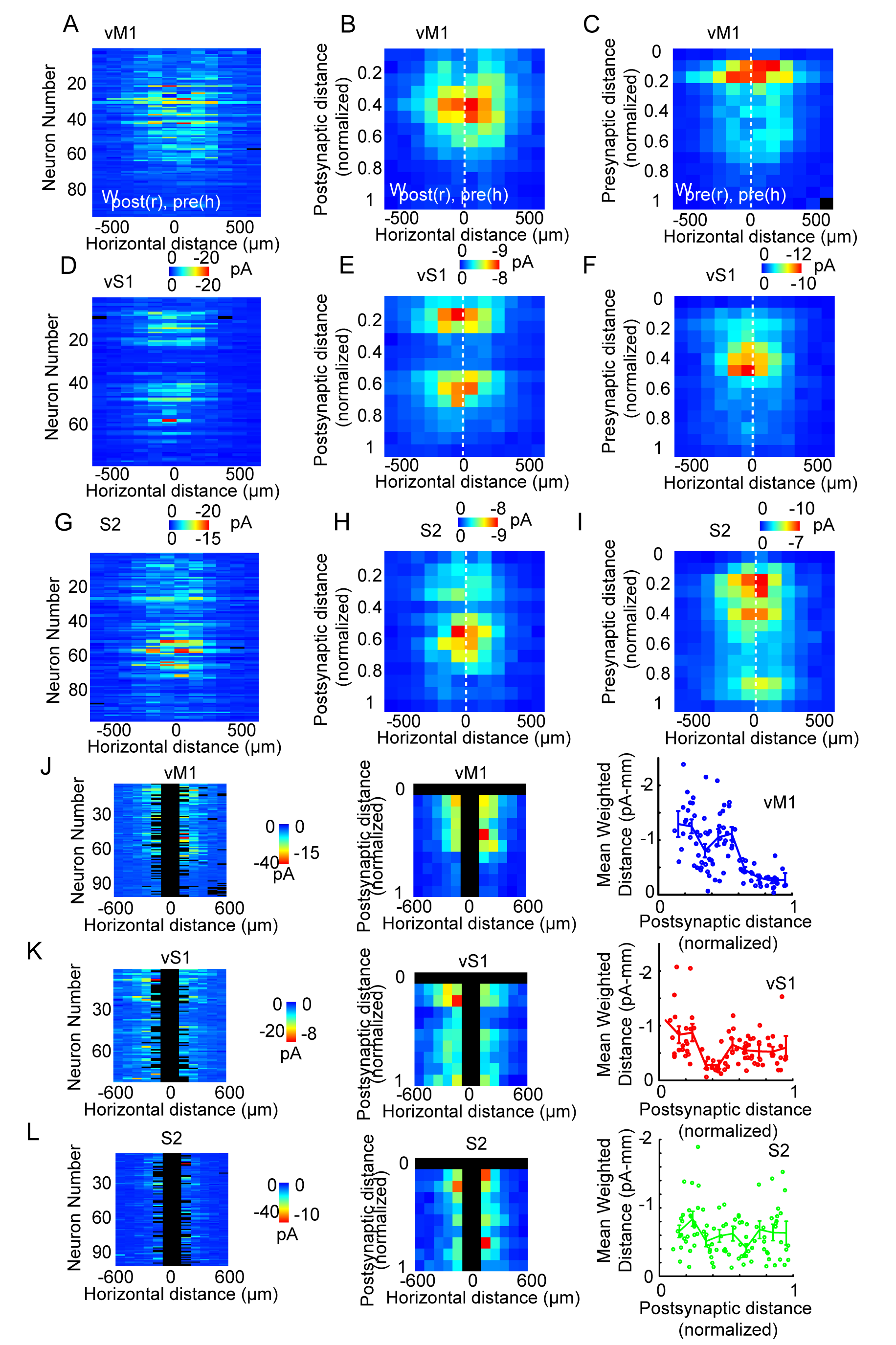

Supplement: Figure S9 — Analysis of horizontal and oblique pathways. (A) Each vM1 neuron's input map was projected onto a vector representing the horizontal profile of synaptic input. The vectors were sorted by postsynaptic position. (B) Same data as for (A), but grouped into distance bins and averaged. Equivalent to projection of the 3-D map data array onto the postsynaptic-horizontal plane. (C) Projection of the 3-D map data array onto the presynaptic-horizontal plane (orthogonal to B). (D–F) Same analyses as (A–C), for vS1 data set. (G–I) Same analyses as (A–C), for S2 data set. (J–L) Horizontal-only analysis. Only “home-layer” (intralaminar; ±0.1 radial distance) data were used to generate each neuron's horizontal vector. For example, top vectors show L2 horizontal inputs to L2. Left: all neurons' vectors, sorted by postsynaptic position. Middle: vectors were grouped into distance-based bins and averaged. Black pixels: sites <100 µm from soma were excluded, and the top bin (corresponding to L1) was empty. Right: Mean weighted distance of individual and averaged horizontal data as a function of postsynaptic position. Values represent the mean, for each neuron, of the vector values times the pixel distances from the soma. Lines with error bars represent mean ± s.e.m. (0.66 MB TIF) [file pbio.1000572.s010.tif]

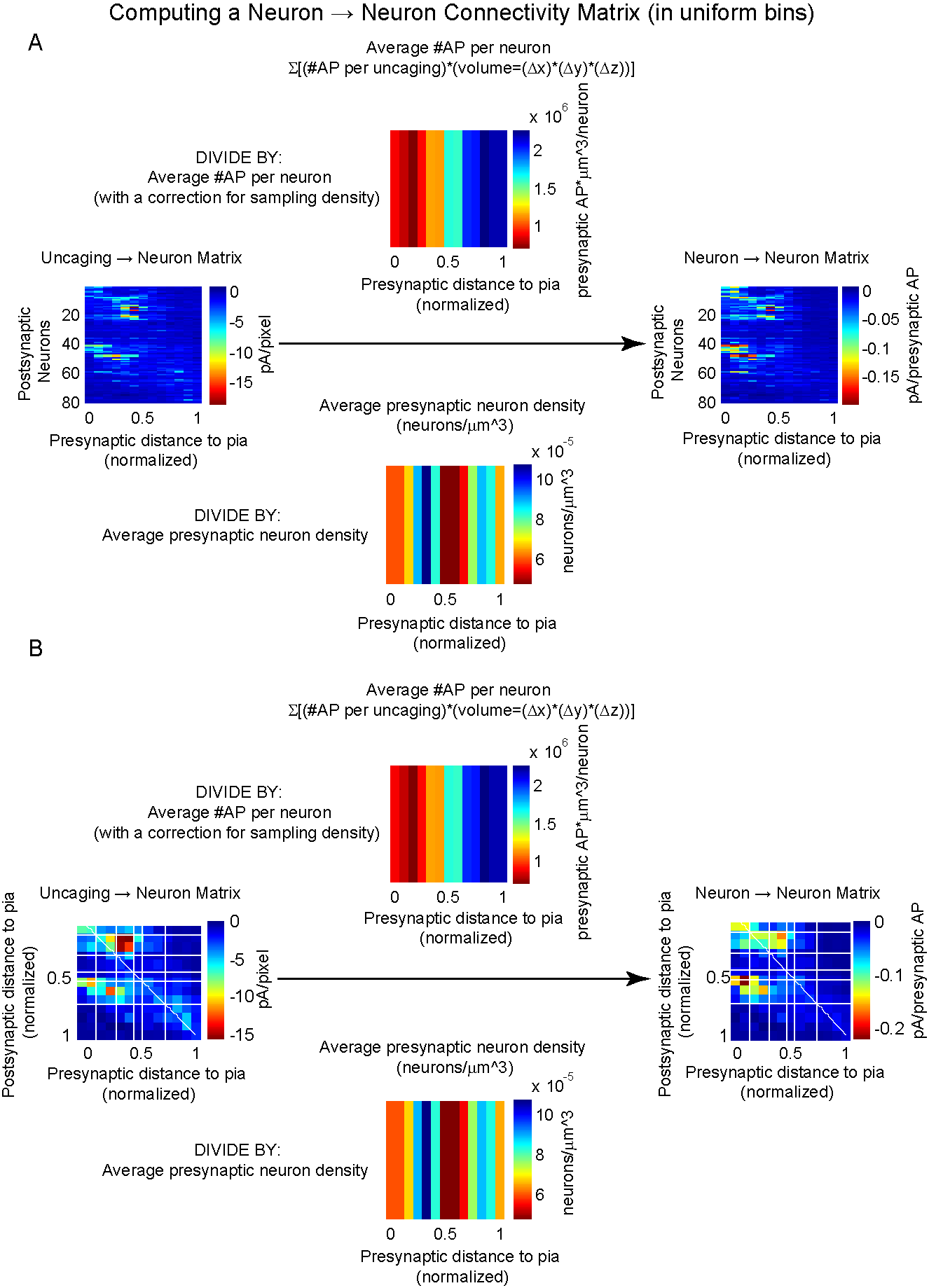

Supplement: Figure S10 — Construction of neuron→neuron connectivity matrices. (A) Example of how neuron→neuron connectivity matrices are constructed; vS1 is used for this example. Given the individual input vectors to a given neuron (Figure 6A–G) averaged in evenly spaced bins, these data are presented for all neurons as an “uncaging→neuron” matrix (left). Cell-type specific excitability is accounted for by dividing each presynaptic bin by the average number of AP per region (top). Corrections are shown as a 2D matrix; corrections are the same for all columns along the presynaptic orientation. Furthermore, input is divided by presynaptic cell density to correct for the number of neurons activated per uncaging event (bottom). Thus, a neuron→neuron connectivity matrix is presented (right). (B) Data presented as in (A), but with postsynaptic neurons binned. Average laminar borders are superimposed. Connectivity matrices in this style are presented in Figures 6 and 7. (0.45 MB TIF) [file pbio.1000572.s011.tif]

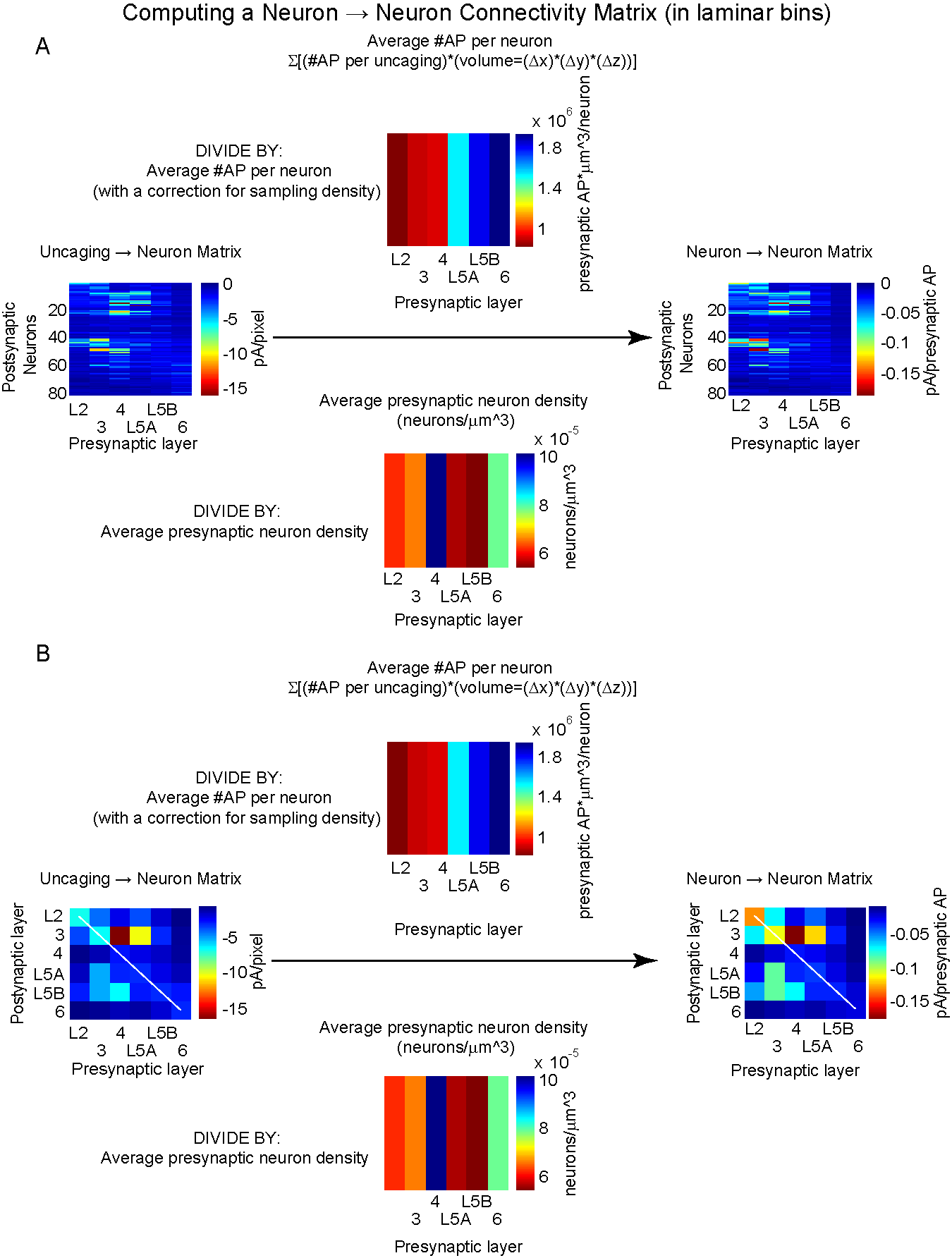

Supplement: Figure S11 — Construction of neuron→neuron connectivity matrices in cortical layer bins. (A) Example of how neuron→neuron connectivity matrices are constructed; vS1 is used for this example. Process is identical to Figure S10, but bins are determined based on boundaries between cortical layers instead of even spacing. Given the individual input vectors to a given neuron (Figure 6A) averaged in laminar specific bins, these data are presented for all neurons as an uncaging→neuron matrix (left). Cell-type specific excitability was accounted for by dividing each presynaptic bin by the average number of AP per region. Corrections are shown as a 2D matrix; corrections are the same for all columns along the presynaptic orientation. Furthermore, input was divided by presynaptic cell density to correct for the number of neurons activated per uncaging event, resulting in a neuron→neuron connectivity matrix (right). (B) Data presented as in (A), but with postsynaptic neurons binned into cortical layer specific bins. (0.39 MB TIF) [file pbio.1000572.s012.tif]

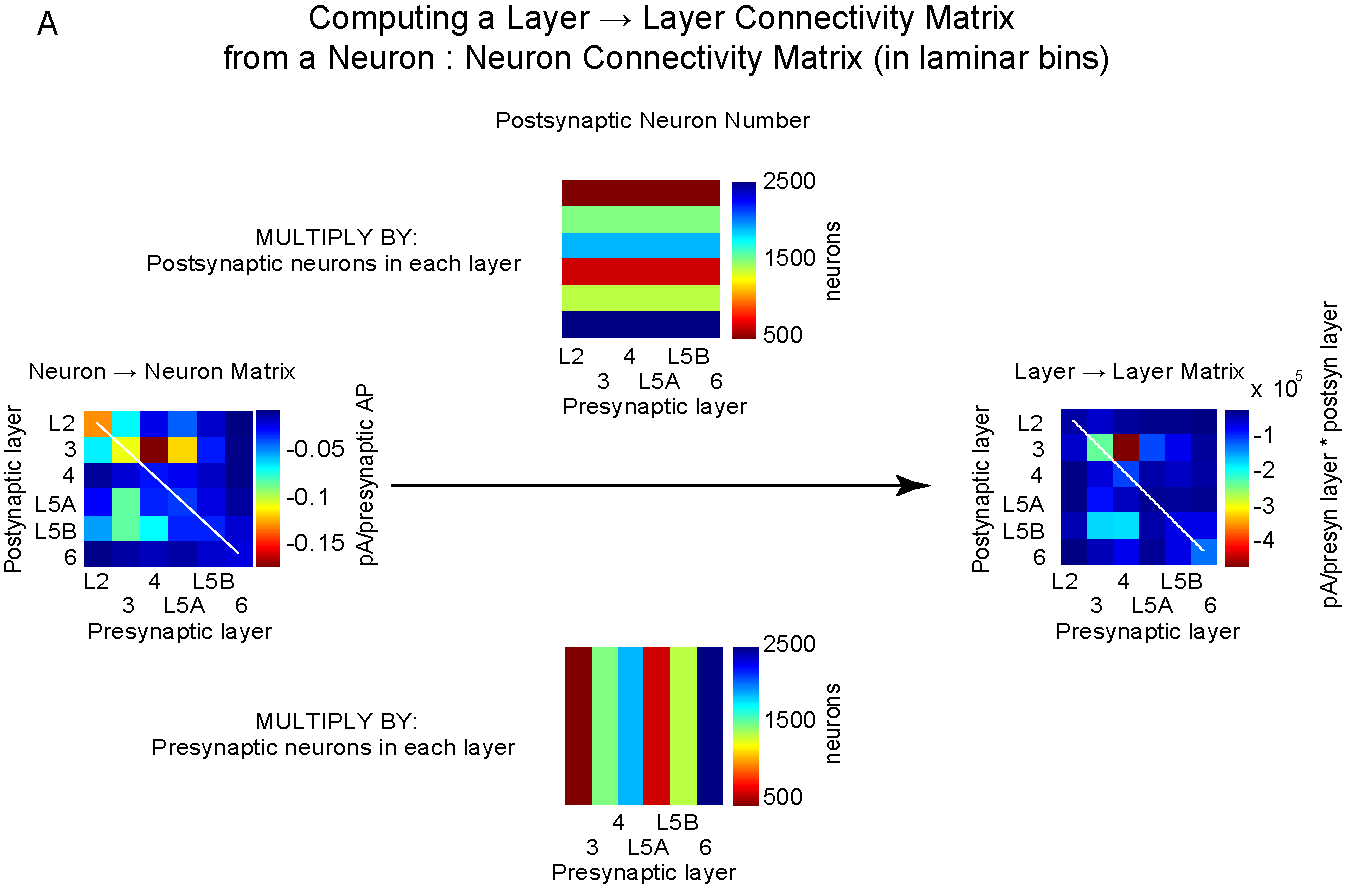

Supplement: Figure S12 — Construction of layer→layer connectivity matrices in cortical layer bins. (A) Example of how layer→layer connectivity matrices are constructed from neuron→neuron connectivity matrices; vS1 is used for this example. The corrected neuron→neuron matrix of Figure S11B (right) is used as a starting point. For the purpose of determining total number of neurons per layer, instead of density, a cortical column of 300×300 µm was used in the plane normal to the radial axis from pia to white matter. The thickness of each layer along the radial axis was based on cytoarchitectonic measurements (Table 1); density was based on Figure S4. Correction to the neuron→neuron matrix involved multiplication by both the number of presynaptic (bottom; columns) and postsynaptic (top; rows) neurons. Connectivity matrices in this style are presented in Figure 7. (0.23 MB TIF) [file pbio.1000572.s013.tif]

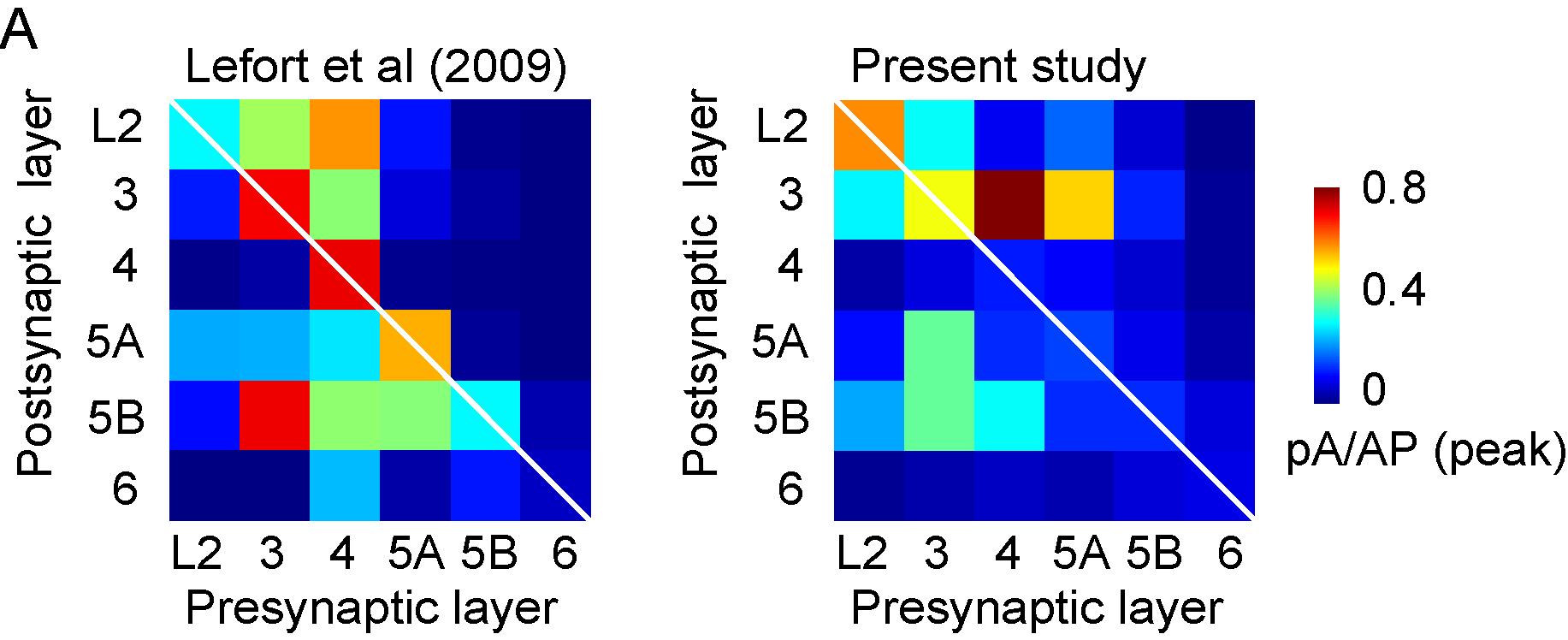

Supplement: Figure S13 — Quantitative comparison of neuron→neuron connectivity derived from complementary methods. (A) Matrices of neuron→neuron connectivity based on pair recordings [27] and LSPS (Hooks et al., this work) plotted on the same scale. Single cell connectivity for pair recordings is converted from peak amplitude in mV to pA using layer specific input resistance, and multiplied by connection probability. Single cell connectivity for LSPS is converted from mean amplitude in pA to peak amplitude using a conversion factor of 0.2 (based on ratio of mean/peak amplitude in LSPS recordings). (0.43 MB TIF) [file pbio.1000572.s014.tif]

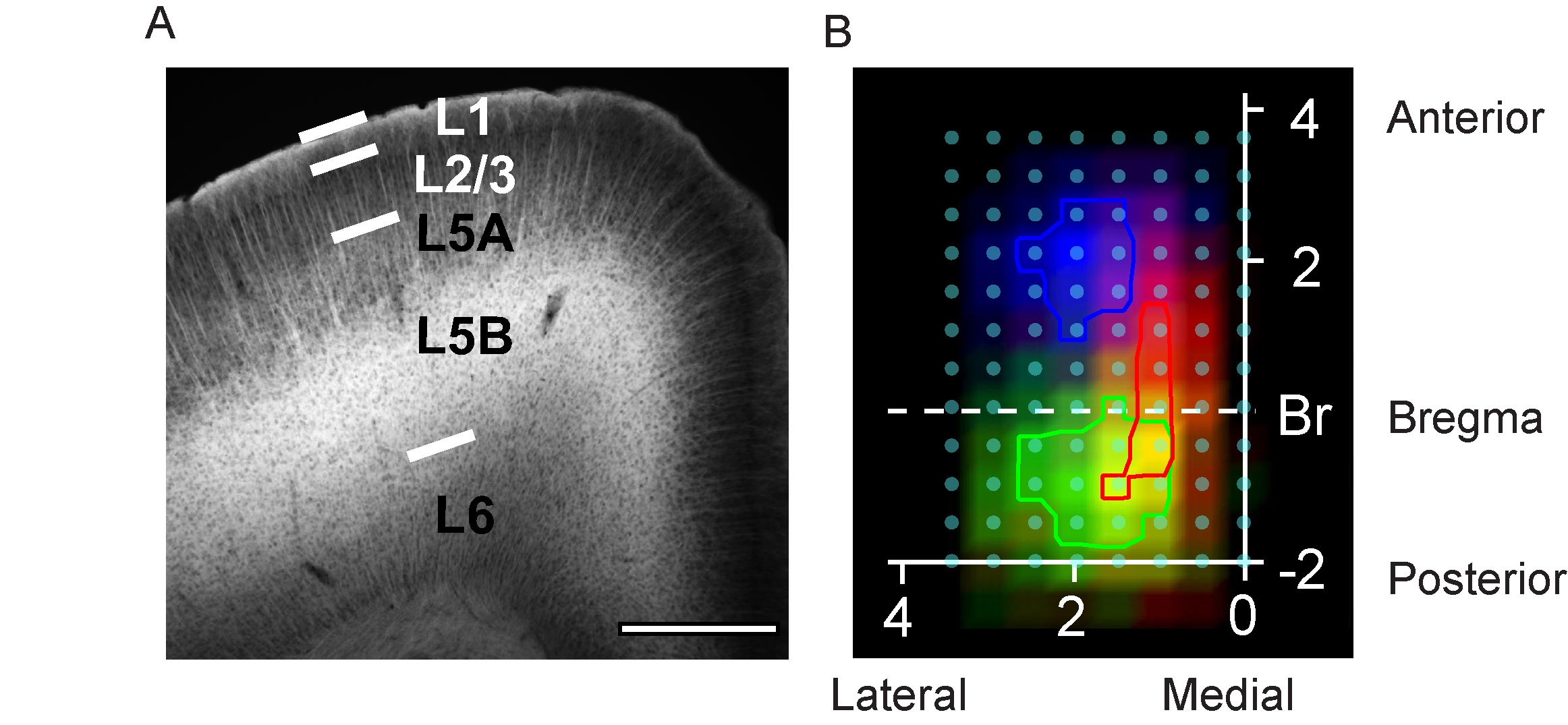

Supplement: Figure S14 — Functional motor mapping using optical microstimulation. (A) Coronal brain slice through motor cortex, prepared from a Thy1-ChR2-YFP (line 18) mouse (scale bar: 0.5 mm). (B) Functional motor map of whisker (red), forelimb (green), and tongue (blue), aligned to bregma (Br) and averaged across six animals. Distances are in mm from the midline or bregma. Colored lines delimit the area where movement could be evoked at threshold power in at least 50% of trials. The grid of the stimulation pattern is indicated with light blue dots (0.5 mm spacing, scale in mm). (2.18 MB TIF) [file pbio.1000572.s015.tif]

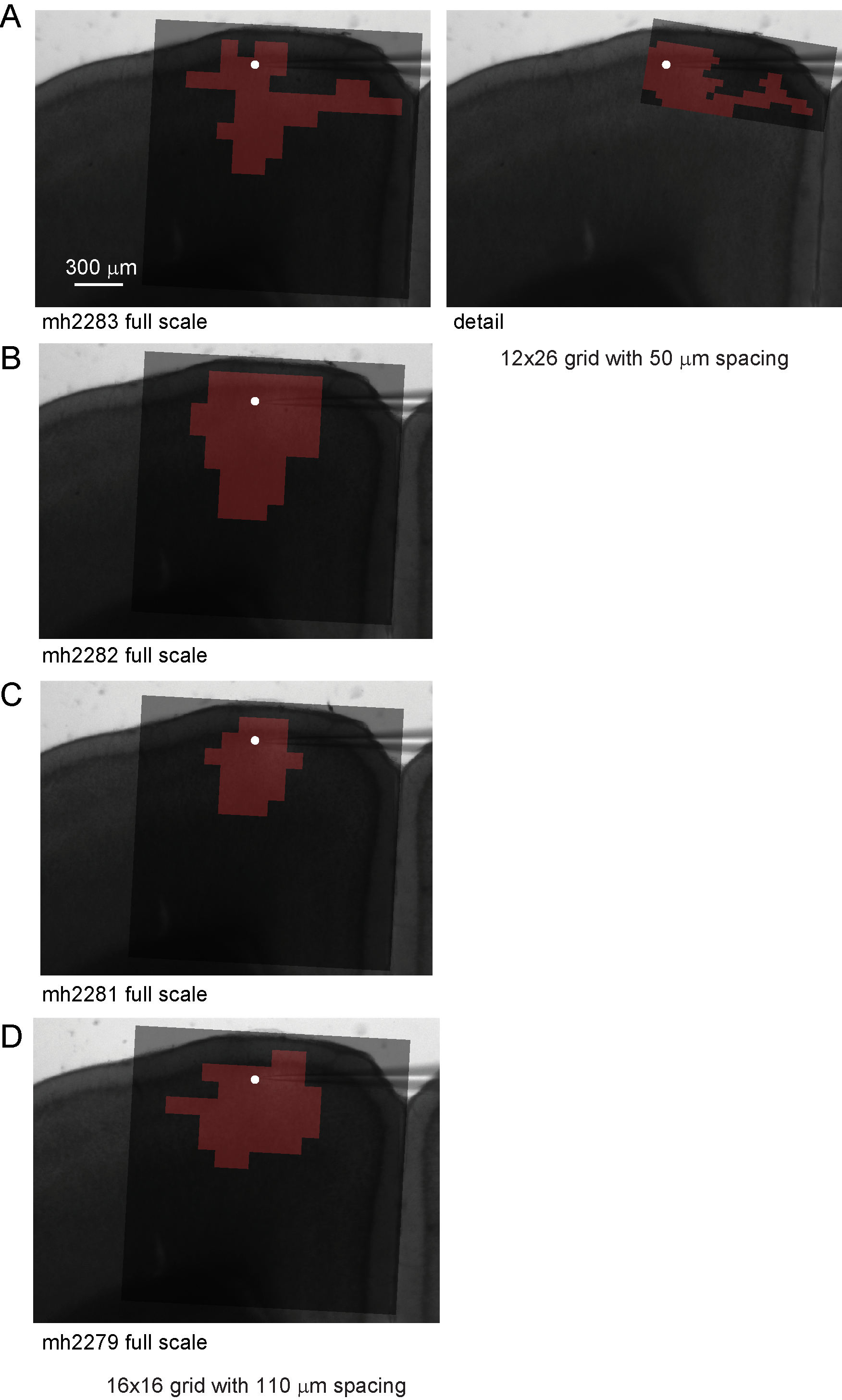

Supplement: Figure S15 — Channelrhodopsin-based mapping of the extent of axonal innervations of local circuits in brain slice. (A) vM1 neurons were transfected with channelrhodopsin-2 by stereotactic injection of adeno-associated virus. vM1 slices were prepared as for LSPS circuit mapping and placed in normal artificial cerebrospinal fluid containing 2 mM Ca2+ and 1 mM Mg2+, as well as 5 µM CPP and 10 µM NBQX to block excitatory synaptic transmission. L2/3 pyramidal neurons were then recorded in cell-attached configuration while exciting the slice with 1 ms pulses of 1 mW 473 nm laser light. Grid spacing was selected to cover a large area (110×110 µm, 16×16) or a detailed area (50×50 µm, 12×26). Points where an action potential was evoked are indicated in red overlay; points without excitation are shown in gray. White circle indicates L2/3 soma. Left panels are large area maps overlaid on the slice image. The right image in (A) demonstrates the spatial resolution (∼50 µm) of the excitation on a medial extending branch of the axon. (B, C, D) Additional examples presented in the same format. (2.80 MB TIF) [file pbio.1000572.s016.tif]

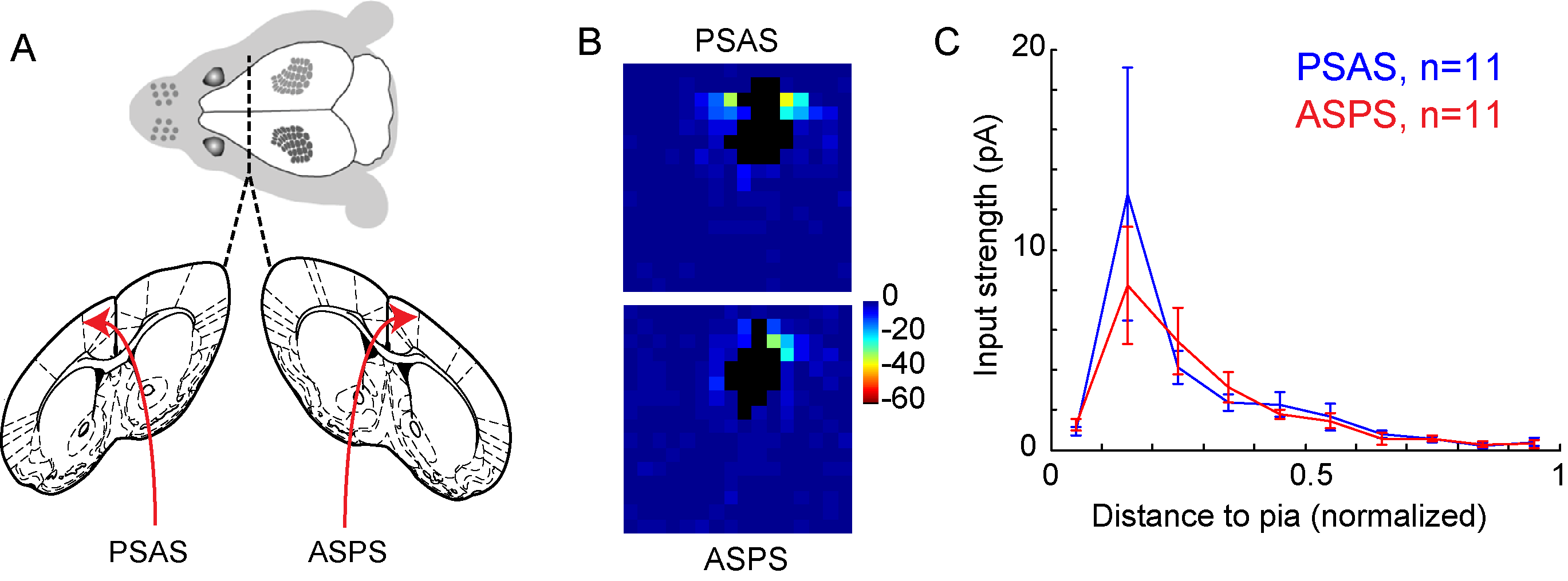

Supplement: Figure S16 — Input mapping of afferents to L5A neurons in vibrissal motor cortex using opposite faces of adjacent brain slices. (A) Experimental design. Two adjacent brain slices were prepared from the same animal. The posterior side of the anterior slice (PSAS) and anterior side of the posterior slice (ASPS) were used for recording (as previously described). (B) Representative input maps from neurons on PSAS (top) and ASPS (bottom) slices. (C) Group data for n = 11 neurons on each side. Average input strength is quantified as the mean of the input vector for each given presynaptic depth bin, and presented ± s. e. m. (0.49 MB TIF) [file pbio.1000572.s017.tif]
