# Supplementary material for: Laminar Analysis of Excitatory Local Circuits in Vibrissal Motor and Sensory Cortical Areas
Source: PLoS Biol. 2011 Jan 4;9(1):e1000572. doi: 10.1371/journal.pbio.1000572 (PMC3014926; doi:10.1371/journal.pbio.1000572)
Supplement: Figure S8 — Comparison of molecular and anatomical definition of cortical lamination. (A, B) Sample in situ images of gene expression from coronal sections of vM1 in the Allen Brain Atlas. Etv1 and Wfs1 illustrated. (C) Brightfield image of vM1 with the axis along which measurements of relative laminar depth were taken indicated. Cortex is marked from 0 (pia) to 1 (white matter). White marks to the right of the ladder indicate cytoarchitectonic boundaries based on the video image. (D) Molecularly defined layers for vM1 plotted for comparison. Radial distance at which Etv1, Wfs1, Rorβ, Plexin D1, Enc1, Abat, and Fezf2 expression were measured based on images in the Allen Brain atlas. Boundaries indicate onset and offset of expression. There were two laminae for PlexinD1 and Enc1. Etv1 expression contained bands of high (superficial) and low (deep) expression. Thy1-ChR2 mouse (line 18) expressed in L5 neurons in motor cortex. Summary table of measurements for vM1 and vS1 are given below. (3.36 MB PDF) [file pbio.1000572.s009.pdf]

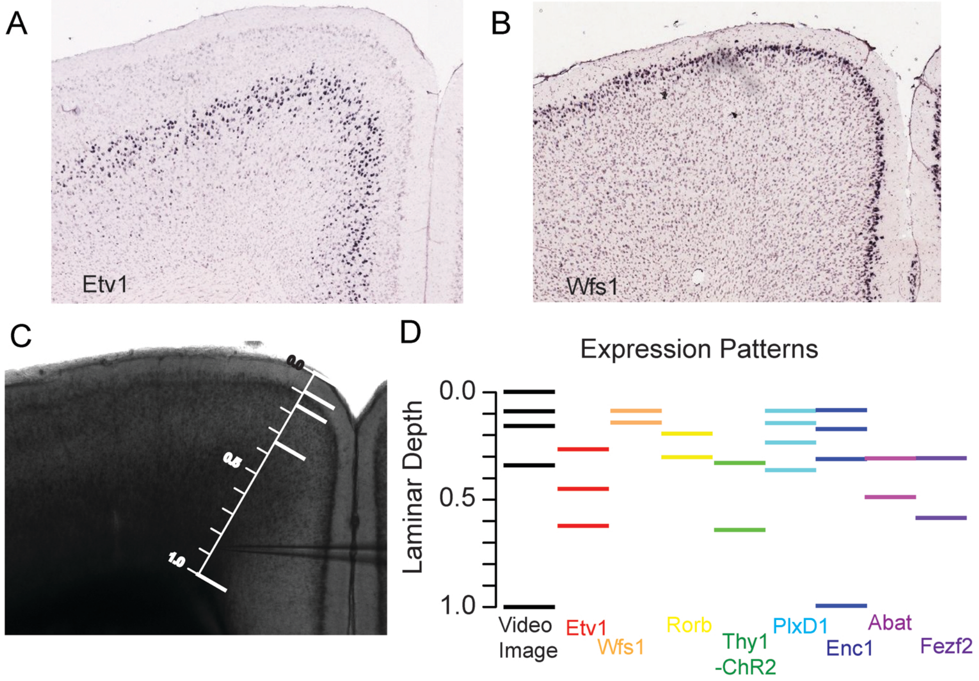

Motor	Brightfield	Etv1	Wfs1	Rorb	Thy1-ChR2	PlexinD1	Enc1	Fezf2	Abat
L1									
	0.09±0.01		0.09±0.01			0.09±0.01	0.08±0.01		
L2									
			0.14±0.01						
L3									
	0.16±0.02	0.26±0.01		0.19±0.01		0.14±0.01	0.17±0.01		
						0.23±0.02			
L5A									
	0.34±0.02	0.45±0.03		0.30±0.02	0.33±0.04	0.36±0.04	0.31±0.03	0.31±0.03	0.31±0.04
L5B									
		0.62±0.03			0.64±0.06			0.59±0.07	0.49±0.04
L6									
	n=5	n=5	n=4	n=5	n=4	n=5	n=5	n=5	n=5

Sensory	Brightfield	Etv1	Wfs1	Rorb	Thy1-ChR2	PlexinD1	Enc1	Fezf2	Abat
L1									
	0.09±0.01		0.08±0.01			0.08±0.01	0.09±0.02		
L2									
			0.14±0.02			0.14±0.01			
L3									
	0.31±0.02			0.20±0.02		0.38±0.02	0.32±0.03		
L4									
	0.46±0.02			0.46±0.03			0.56±0.01	0.49±0.03	0.47±0.02
L5A									
	0.54±0.02	0.51±0.01			0.58±0.02	0.54±0.02		0.67±0.02	0.66±0.02
L5B									
	0.74±0.03	0.70±0.02			0.77±0.02		0.72±0.02		
L6									
	n=62	n=4	n=4	n=5	n=4	n=4	n=4	n=5	n=5
