# Supplementary material for: Laminar Analysis of Excitatory Local Circuits in Vibrissal Motor and Sensory Cortical Areas
Source: PLoS Biol. 2011 Jan 4;9(1):e1000572. doi: 10.1371/journal.pbio.1000572 (PMC3014926; doi:10.1371/journal.pbio.1000572)
Supplement: Text S1 — Laminar analysis of excitatory local circuits in vibrissal motor and sensory cortical areas. Supplemental information to the article. Sections include: Supplemental Methods (relationship of pixel values in input maps to average synaptic connection strength (q con); converting input maps to connectivity matrices; methods for optical microstimulation), Supplemental Discussion (comparison and limitations of circuit mapping techniques; horizontal connectivity), and References. (0.32 MB RTF) [file pbio.1000572.s019.rtf]

Laminar analysis of excitatory local circuits in vibrissal motor and sensory cortical areas

Running title: Comparative circuit mapping in vM1, vS1, and S2

B. M. Hooks1,2, S. Andrew Hires1, Ying-Xin Zhang1,3, Daniel Huber1, Leopoldo Petreanu1, Karel Svoboda1, Gordon M. G. Shepherd1,2
1Janelia Farm Research Campus, Howard Hughes Medical Institute, Ashburn, VA 20147; 2Department of Physiology, Feinberg School of Medicine, Northwestern University, Chicago, IL 60611
3The Solomon H. Snyder Department of Neuroscience, Johns Hopkins School of Medicine, Baltimore, MD 21205

SUPPLEMENTAL METHODS
Relationship of pixel values in input maps to average synaptic connection strength (qcon)
The elements, qcon, in the neuron→neuron connectivity matrix are related to LSPS input maps:
	

where pixel value is the average value (in pA) of the postsynaptic response to a single uncaging event at a single point in an input map during the synaptic response window (7-50ms following uncaging), ñcell is the neuronal density at the point of uncaging (neurons/µm3), Vexc is the volume of excited neurons (µm3), SAP is the number of action potentials (APs) fired per presynaptic neuron (AP/neuron), and qcon is the average strength of the synaptic connection per AP in a presynaptic neuron (pA/AP). 
The term qcon conflates connection probability and unitary connection strength:
	

where pcon is the connection probability between a given presynaptic location and a postsynaptic location anduEPSC is the unitary EPSC strength (in pA/AP) for a single AP in a presynaptic neuron connected to a postsynaptic neuron.
The total number of APs per uncaging event, NAP, is related to the photoexcitability parameters: 
	

Converting input maps to connectivity matrices
We derive values for the connectivity matrix using pixel values from input maps.  We correct pixel values for cell density and excitability (combined into NAP) to extract qcon from input map values.  Figure S4 explains our methodology for computing ñcell.  It is difficult to directly measure Vexc  and SAP.  Instead, we quantified the total number of APs during maps that were performed in cell-attached configuration (excitation profiles).  Laser power and extracellular solutions were identical to those used during whole cell recordings.  These maps were centered on the soma of the recorded neuron and were 8×8 square maps with 50 µm spacing between points.  We quantified the number of action potentials evoked within a 100 ms window following uncaging.  We determined the total number of action potentials per uncaging event, NAP , on a bin by bin or a layer by layer basis by the following formula:

	

where #AP per uncaging site is measured in AP/neuron, from the average excitation profile map for the given location (AP/neuron), Äxmap and Äymap are the spacing of the excitation profile map points (µm; 50 µm was used during experiments), and Äz is the depth in the z-axis excited by uncaging (µm; 100 µm was used for calculation).
Thus, when integrating the total number of AP per excitation profile, the x-,y-, and z- parameters estimate the volume considered.  We estimated 100 µm for the depth of the slice excited to account for attenuation of the UV laser.  We examined the excitability of a subset of neurons in L3 and L5B of vS1 to determine how deep in the slice UV uncaging could excite neurons (Figure S3).  We found a decay in excitability with depth that corresponded to that observed in LSPS in rat under similar conditions [8].  L5B neurons were more excitable than L3 neurons, but excitability of both decreased with slice depth at a similar rate.  No neurons deeper than 100 µm firing action potentials during mapping (though these cells were confirmed to be excitable using higher powers or longer UV exposures); thus this value was used to estimate the volume excited.
We smoothed the excitability correction factor by averaging each bin's results with the adjacent bins shallower and deeper in cortex.  Bins of a standard width (14 bins, each 1/14th of cortex) were used for neuron→neuron connectivity matrix calculations (less than our sampling density of 16 points since 1-2 points may fall below white matter); layer based binning (uneven spacing) was used to compute the layer→layer connectivity matrix.  Since this correction factor, NAP, depends upon presynaptic factors, it appears in columns in our correction process (Figures S10 and S11); in these figures, ñcell, is shown separately.  The uncaging→neuron matrix is corrected as follows:
	

To convert from a neuron→neuron matrix to a layer→layer matrix, it is necessary to multiply the amount of excitation by the number of pre- and post- synaptic cells (Figure S12).  The number of cells in each layer was computed from the density (Figure S4), assuming a 300 µm square column of cortex, with layer thickness based on cytoarchitectonic measurements (Table 1).  We then multiplied the existing neuron→neuron matrix by these total numbers (Figure S12; note that the pre- and post- synaptic corrections being multiplied are orthogonal to each other) to give a layer based estimate of connectivity.  This representation favors neuron-dense and wider cortical layers, and is presented in Fig. 7C, 7F, and 7I.
As a control for differences in the spatial resolution of excitation between layers, we quantified the mean weighted distance to APs in excitation profiles.  Because the map was centered on the soma, the nearest possible distance from the soma to the center of the uncaging beam was 35 µm from the soma.  The mean weighted distance from the soma to action potential generating sites was Ó(APs × distance from soma) / Ó(APs).
Since the mean weighted distance was similar between conditions (Fig. 2D), we did not make corrections for small differences in the resolution of LSPS between layers of cortex or between cortical areas.  

Methods for optical microstimulation
Optical microstimulation was carried out in Thy1-ChR2-YFP (line 18) as described previously [1,2]. The skull over the motor cortex was thinned and covered with a layer of transparent cyanoacrylate glue. During the microstimulation the mice were lightly anesthetized with ketamine/xylazine. A series of 5 pulses (5ms, 20Hz) from a blue laser (470 nm, 150 µm beam size, CrystaLaser) was applied on a 10×8 grid pattern with 500 µm spacing. The beam position on the skull was controlled using galvanometric mirrors (6210H, Cambridge Scanning) and referenced to bregma. Whisker, paw and tongue-movement were recorded with a high speed camera at 200 Hz (Dragonfly Express, Point Gray Research) and analyzed offline. Threshold power was determined by the minimal power necessary to evoke movement in 100% of trials. The boundary of the threshold maps were determined by locations where movement could be evoked at threshold laser power.


SUPPLEMENTAL DISCUSSION
Comparison and limitations of circuit mapping techniques
Several electrophysiology-based techniques, each with their own biases, have been used to map functional connections in neocortical brain slices, with overlapping results. These include LSPS, pair recording, and channelrhodopsin-2 assisted circuit mapping (CRACM).  Limitations of LSPS have been discussed previously [9].
The use of whole-cell recording for circuit mapping has inherent limitations. For one, it provides a soma-centric view of input strength, and measurements of synaptic strength may be distorted by dendritic attenuation and incomplete voltage control [5,6]. Local depolarization in distal dendrites may activate voltage-gated conductances, and these active conductances may vary between different classes of inputs. Passive dendritic filtering will attenuate distal inputs more than proximal ones. These systematic errors are notoriously difficult to estimate.
In slice preparations, some inputs are lost due to truncation of axons and dendrites.  Longer-range pathways are relatively more attenuated by slicing than shorter-range pathways, biasing connectivity matrices [10].  Slice artifact may vary with slice angle and brain region.  The slice angles used in this study were chosen to preserve apical dendritic trunks of pyramidal neurons, and also the main trunk of the descending axon. Vertical (Figures 3-5 and S5-S7) and horizontal connectivity (Figures S5-S7 and S9) spanned distances of 1 mm or more, comparable to the thickness of the cortex, suggesting that strong circuits were readily detected. 
LSPS measurements are perturbed by strong direct responses from dendrites of the recorded neurons, causing an underestimate of local, mainly intralaminar connections relative to pair recordings.  For example, our methods undersample the dense connections known to occur between L4 neurons within a barrel.  However, LSPS mapping rapidly and efficiently samples many connections. Thus, approximately 100 recordings are sufficient to map the connections between cortical laminae.  This variation in efficiency is an essential tradeoff, which makes LSPS effective for a comparative study of cortical regions.
	Pair recordings involve exciting a single presynaptic cell and searching for unitary synaptic responses in postsynaptic neurons.  Pair recordings provide unitary event amplitudes, short term dynamics, and connection probabilities.  They are relatively labor-intensive as thousands of pair recordings are required to map the circuits between cortical layers in one area [7].  Pair recordings suffer from some of the same limitations that affect other slice recording methods, including the cutting of an entire connection, or reducing the amplitude of a unitary synaptic connection by severing a portion of the axon (or dendrite).  Furthermore, data sets are potentially skewed towards high probability connections (e.g. between nearby neurons), thus potentially undersampling the full range of horizontal offset.  Longer-range connections are especially challenging to study quantitatively because low connection probabilities reduce the number of connected pairs sampled.  
In CRACM [3,4], groups of ChR2-positive neurons or axons are excited simultaneously.  CRACM allows detection of synapses across all length scales, since excitation of ChR2-positive axons is possible even if the axon is severed from the parent soma. But it is difficult to estimate the number of activated axons. CRACM thus provides only relative values for connection strength between a particular activated set of axons and different postsynaptic populations.  Comparisons across different experiments, in which different populations of axons are activated, usually rely on normalization methods. 

Effect of slice artifact on local connectivity
Because of the possibility that our slice preparation cuts axonal arbors, we performed control experiments to verify the distance over which axons were intact in our slice preparation (Figure S15). We infected neurons in a defined cortical region by stereotactic injection of an adeno-associated virus expressing ChR2.  We prepared brain slices and recorded from ChR2-positive neurons using loose-seal cell-attached recordings. CPP and NBQX were added to prevent feed-forward excitation between neurons.  We stimulated axonal arbors in a grid pattern on the slice using a short pulse of blue light.  We examined locations within the slice at which stimulation resulted in action potentials propagation from the axon into the soma.
In all cases (n=4) axons arborize extensively in L2/3, L5A, and in some cases L5B.  The axon extends for several hundred micrometers (up to 1 mm), larger than the spatial extent of connectivity.  If the neuron from which we recorded were excited in an equivalent LSPS experiment, then synaptic excitation could be transmitted to neurons whose dendrites fall in the excitable area.  
Furthermore, we examined the degree to which slice angle affected the strengths of certain pathways in our slice preparation.  We selected two adjacent (bookmatched) off-coronal (see Methods) slices and recorded from either the posterior side of the anterior slice, or the anterior side of the posterior slice.  Thus we recorded from adjacent surfaces of the cortex separated during cutting (Figure S16A).  We then recorded input maps from L5A neurons, showing overlapping distributions of input strength for the two sides (Figure S16C).  From this we conclude that slice angle did not affect the strongest descending pathway in vM1.  Nor did distinct pathways from those previously identified appear upon using the slice facing in the opposite direction.

Horizontal connectivity
	We also analyzed horizontal connectivity to assess variations in the strength of input outside of a neuron's home column.  This involved averaging input maps into vectors corresponding to horizontal locations offset medially and laterally from the postsynaptic neuron (pre(h)), instead of averaging by presynaptic radial position (pre(r)).  This generated matrices with axes of radial postsynaptic and horizontal presynaptic positions; i.e., Wpost(r), pre(h), which we display as both vectors for individual cells (Figure S9A for vM1) or binned versions (Figure S9B).  In a mathematically similar manner, we were able to generate Wpre(r), pre(h) and display matrices with axes of presynaptic radial and presynaptic horizontal positions (Figure S9C).  These plots showed input that was generally centered around, and maximal nearest, points along the same radial axis as the postsynaptic neuron.  For vM1, these matrices showed that the inputs are distributed horizontally over a wider distance in shallow layers.  The horizontal extent of vS1 input was roughly 200-300 µm wide on either side, similar to S2, despite the absence of barrels.

REFERENCES
1. Komiyama T, Sato TR, O'Connor DH, Zhang YX, Huber D, et al. (2010) Learning-related fine-scale specificity imaged in motor cortex circuits of behaving mice. Nature 464: 1182-1186.
2. Ayling OG, Harrison TC, Boyd JD, Goroshkov A, Murphy TH (2009) Automated light-based mapping of motor cortex by photoactivation of channelrhodopsin-2 transgenic mice. Nat Methods 6: 219-224.
3. Petreanu L, Huber D, Sobczyk A, Svoboda K (2007) Channelrhodopsin-2-assisted circuit mapping of long-range callosal projections. Nat Neurosci 10: 663-668.
4. Petreanu L, Mao T, Sternson SM, Svoboda K (2009) The subcellular organization of neocortical excitatory connections. Nature 457: 1142-1145.
5. Spruston N, Jaffe DB, Williams SH, Johnston D (1993) Voltage- and space-clamp errors associated with the measurement of electrotonically remote synaptic events. J Neurophysiol 70: 781-802.
6. Williams SR, Mitchell SJ (2008) Direct measurement of somatic voltage clamp errors in central neurons. Nat Neurosci 11: 790-798.
7. Lefort S, Tomm C, Floyd Sarria JC, Petersen CC (2009) The excitatory neuronal network of the C2 barrel column in mouse primary somatosensory cortex. Neuron 61: 301-316.
8. Shepherd GMG, Svoboda K (2005) Laminar and columnar organization of ascending excitatory projections to layer 2/3 pyramidal neurons in rat barrel cortex. J Neurosci 25: 5670.
9. Weiler N, Wood L, Yu J, Solla SA, Shepherd GM (2008) Top-down laminar organization of the excitatory network in motor cortex. Nat Neurosci 11: 360-366.
10. Stepanyants A, Martinez LM, Ferecskó AS, Kisvárday ZF (2009) The fractions of short- and long-range connections in the visual cortex. Proc Natl Acad Sci U S A 106: 3555-3560.
